# CATCH-IT Report: Evaluation of an Internet-Based Smoking Cessation Program: Lessons Learned From a Pilot Study

**DOI:** 10.2196/jmir.6.4.e47

**Published:** 2004-12-31

**Authors:** Cameron Norman

**Affiliations:** ^2^Vancouver Coastal Health Research UnitVancouver General HospitalUniversity of British ColumbiaVancouver BCCanada; ^1^Centre for Global eHealth InnovationUniversity Health NetworkUniversity of TorontoToronto ONCanada

**Keywords:** Internet, smoking cessation, health behaviour, World Wide Web, computers, technology

## Introduction

It is estimated that more than 4 million people die annually from tobacco-related illnesses globally [1]. Well-designed smoking prevention and smoking cessation programs can make substantial contributions to global public health. Any intervention that can reduce tobacco use, offer global reach, and do so in a cost-effective manner presents a tremendous opportunity to reduce the future burden of disease. To this end, the paper by Feil et al, *Evaluation of an Internet-based smoking cessation program: lessons learned from a pilot study* [2] represents a significant opportunity to move tobacco control forward. This paper reports on a pilot test evaluation of a Web-based smoking cessation program, the Quit Smoking Network (http://www.qsn.ori.org/), and the efforts to recruit participants and conduct a study through the Internet.

The authors of this paper have considerable experience in medical computing and in the burgeoning area of behavioural eHealth, having published several recent papers on Internet-based interventions for diabetes [3-6]. Although this is the first of their papers to address smoking cessation, the authorship team includes a recognized expert in the field of tobacco control (Ed Lichenstein) who also plays a role in the delivery of the intervention. The site is publicly available; however, it was unavailable for viewing at the time of this review because of upgrades to the website.

## Objectives

The aim of the study is to evaluate a strategy for online study recruitment and retention, to evaluate the influence of incentives on follow-up response, and to assess the impact of the Quit Smoking Network site on smoking behaviour.

Feil and colleagues [2] describe their intervention as having several components including

a structured intervention that guides development of a cessation quit plan, interpersonal support (both peer-peer and professional-peer support in postings forum and E-mail response formats), and a library of a wide variety of cessation resources (e.g., online pamphlets, motivations materials, and links to other sites).

The intervention was also described as "based on theoretically grounded and empirically validated intervention approaches" citing an earlier paper by Lichenstein and Glasgow [7]. Despite this assertion, no reference to a specific theoretical model for the intervention or theory of implementation was provided (see Grembowski, 2001 [8]). Social support and self-efficacy are mentioned as desired process outcomes, but how these theories were used is not made explicit.

## Methods

The research design is a single-condition study with a randomized follow-up component. Participants were recruited largely through magazine ads, local media coverage of the study and through the website itself (hits, web searches etc). All participants were exposed to the website intervention and then randomly assigned to 1 of 4 follow-up conditions (2 email and 2 postal mail) afterwards. Participants completed a pretest survey online prior to the intervention and were contacted at 3 months postintervention to complete the follow-up online. Participants who did not complete the 3-month follow-up were randomly assigned to receive further follow-up notifications by email or regular US mail after 3 weeks. No description of the randomization process was provided.

## Results

Two hundred and nine participants (56%) completed the 3-month follow-up, mostly through the Internet (81% Web, 5.5% email). Both the mode of communication (US Mail, 60%; email, 55%) and incentive amount (US$20, 60%; US$10, 55%) provided similar rates of follow-up. With regard to the impact of the intervention, 38% (67) of participants reported abstinence (7-day point prevalence) at 3-month follow-up (18% using intent-to-treat analysis). Such results are comparable to many other non-Internet smoking cessation trials [9].

## Limitations

Although there was a reported cessation rate of 18% at 3 months, it is unclear whether this effect can be attributed to the intervention as no method of accounting for alternative explanations was provided. Since participants were self-selected based on an expressed interest in smoking cessation it seems reasonable that participants also sought other treatment options at the time of their participation. Another limitation of the study is the absence of the reported mean time to response after the second 3-month reminder was sent to participants.

From a tobacco control perspective, there are additional concerns. An absence of a detailed description of instruments used to assess smoking variables is unfortunate. With a large body of literature in tobacco control, a number of acceptable measures or items have emerged to assess smoking behaviours and tobacco use. However no details of the items, item source, or scale reliability were provided and, in the case of cessation self-efficacy, were not even defined. Outside the effect on cigarette abstinence, the study's effects on outcome variables such as cessation self-efficacy were not reported.

## Discussion

This study introduces a number of innovations for advancing knowledge of eHealth. The implementation and evaluation of an intervention completely at a distance represents a significant step forward in advancing eHealth research. Another innovation is the study of both incentive value and mode of contact on follow-up participation rates--issues that clearly require further study.

Although the study design was innovative, the study as reported was problematic in a number of areas. With respect to eHealth issues, many of the evaluation reporting guidelines recommended by the Science Panel on Interactive Health Communication [10] were not followed. Furthermore, many basic reporting guidelines from the CONSORT statement were also not followed including a description of the randomization process [11]. Although the study was submitted as a “Brief Report”, it was expected that these quality issues would be mentioned in the text or with reference to another source (eg, web page). Such information enables a reader to assess the study's merit while providing guidance on developing future eHealth intervention studies.

Despite its limitations, many of which could have been reduced by more complete reporting, this is an important study for eHealth and tobacco control. As with many pioneering studies, this work offers more questions for eHealth research than answers; but the answers it does provide are nonetheless important. Furthermore, the findings of the study have great clinical significance for tobacco control given that the intervention was delivered in absentia and the potential for widespread, population-based translation of the intervention is high. Building on the results of this pilot test it is hoped that the authors will soon offer a more extensive evaluation of the Quit Smoking Network, one that has addressed some of the concerns stated here and that furthers this study's unique contribution to the literature.

## Questions for Authors

- What method or process was used to randomize participants into each condition?
- Among those participants who did not respond to the initial follow-up request, what was the mean time to follow-up?
- What are the theoretical model(s) guiding the Quit Smoking Network site and how are they applied?
- How was intervention exposure (dose) assessed or measured for each participant?
- What other smoking strategies did participants report engaging in? If not measured, why?
- How was cessation self-efficacy measured?
- What were the levels of reported social support at baseline and how were such measures correlated with smoking cessation at 3-month follow-up?
- How do you propose researchers address the issue of validating smoking self-report using remotely-delivered interventions?

## Multimedia Appendix

Powerpoint slides by C. Norman presenting the Feil paper.
