# Supplementary material for: CATCH-IT Report: Evaluation of an Internet-Based Smoking Cessation Program: Lessons Learned From a Pilot Study
Source: J Med Internet Res. 2004 Dec 31;6(4):e47. doi: 10.2196/jmir.6.4.e47 (PMC1550626; doi:10.2196/jmir.6.4.e47)
Supplement: Supplementary file 1 [file jmir_v6i4e47_app1.ppt]

## Slide 1
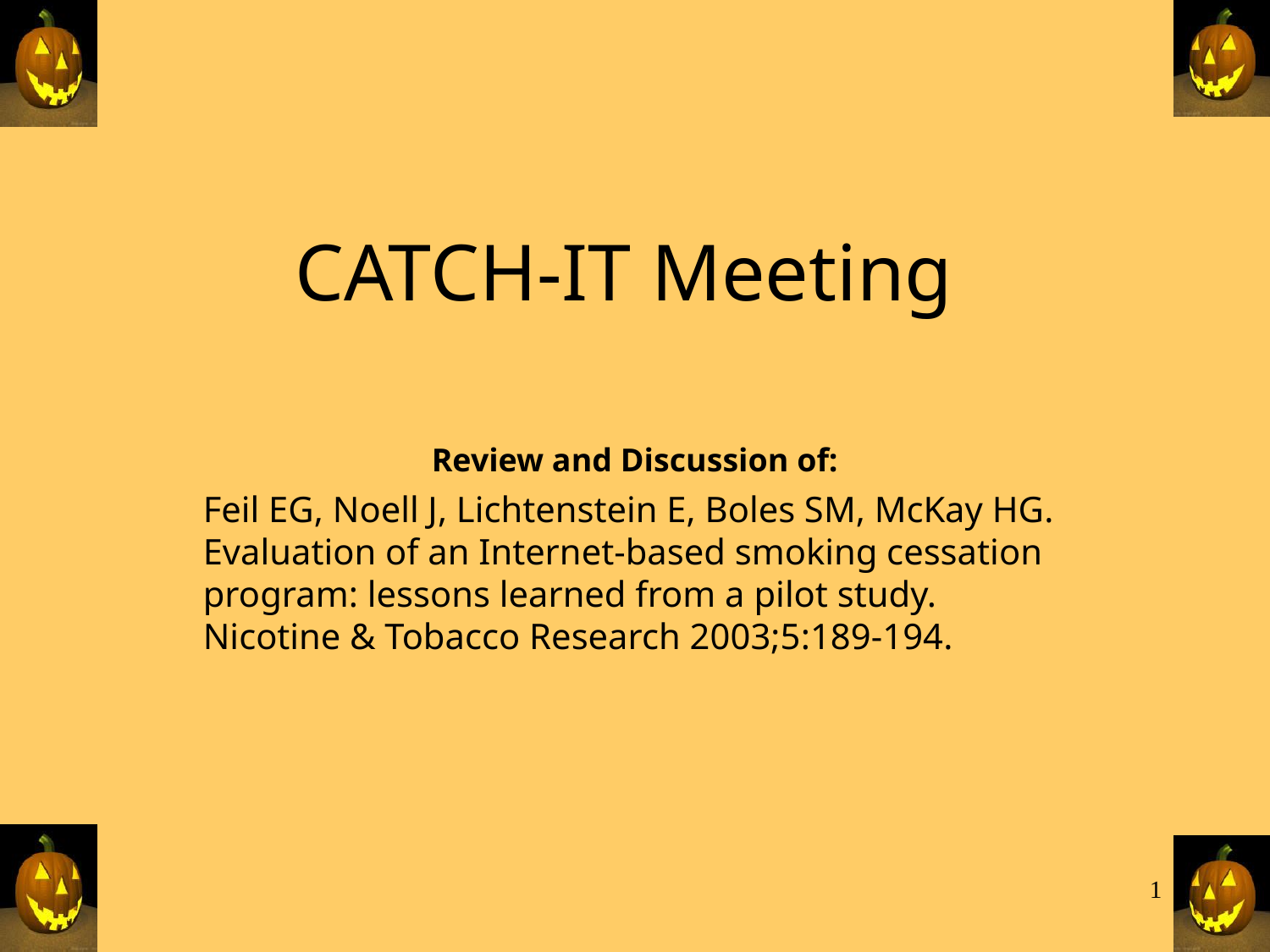

# CATCH-IT Meeting
Review and Discussion of:
Feil EG, Noell J, Lichtenstein E, Boles SM, McKay HG. Evaluation of an Internet-based smoking cessation program: lessons learned from a pilot study. Nicotine & Tobacco Research 2003;5:189-194.
1

## Slide 2
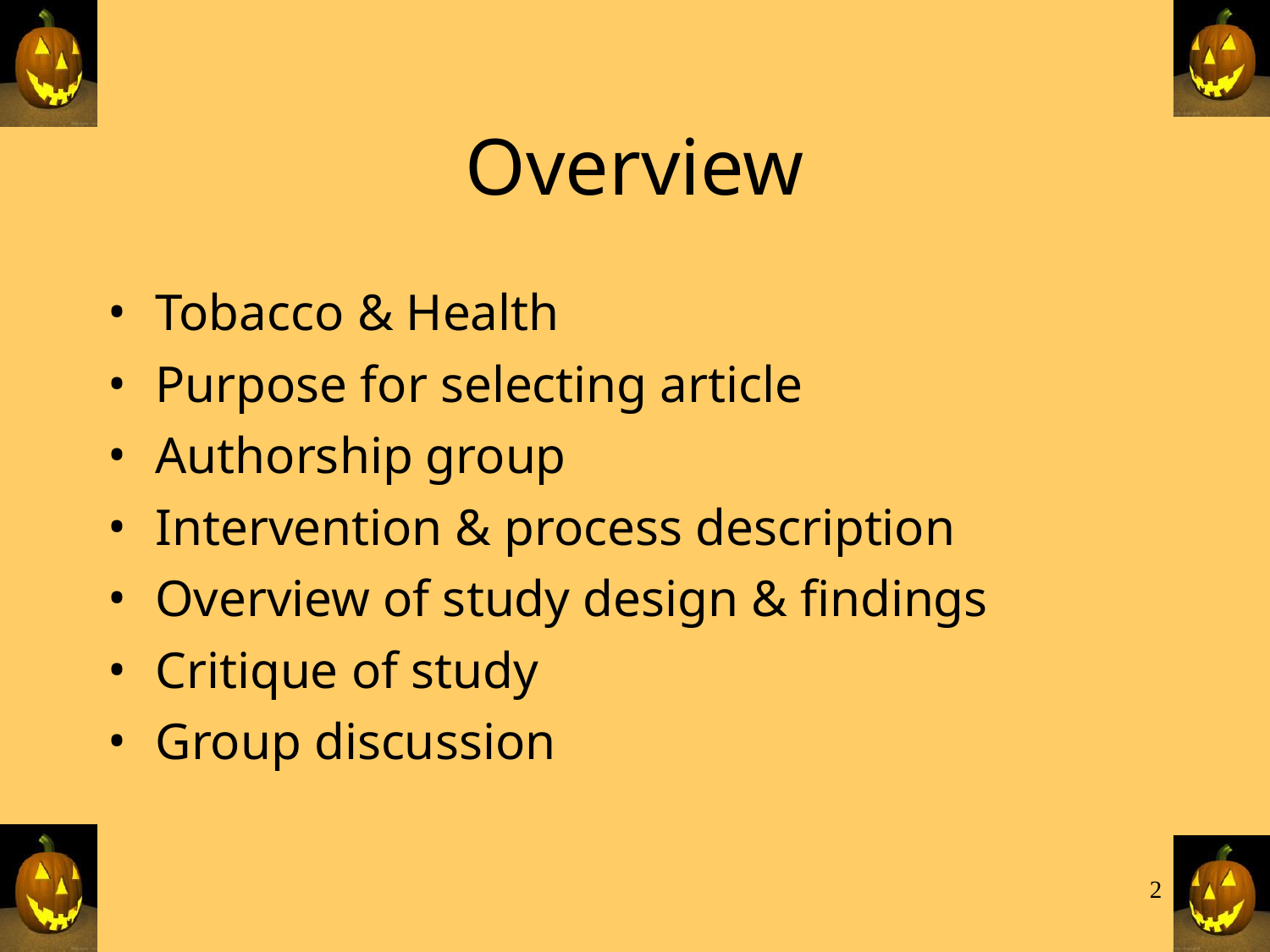

# Overview
Tobacco & Health
Purpose for selecting article
Authorship group
Intervention & process description
Overview of study design & findings
Critique of study
Group discussion
2

## Slide 3
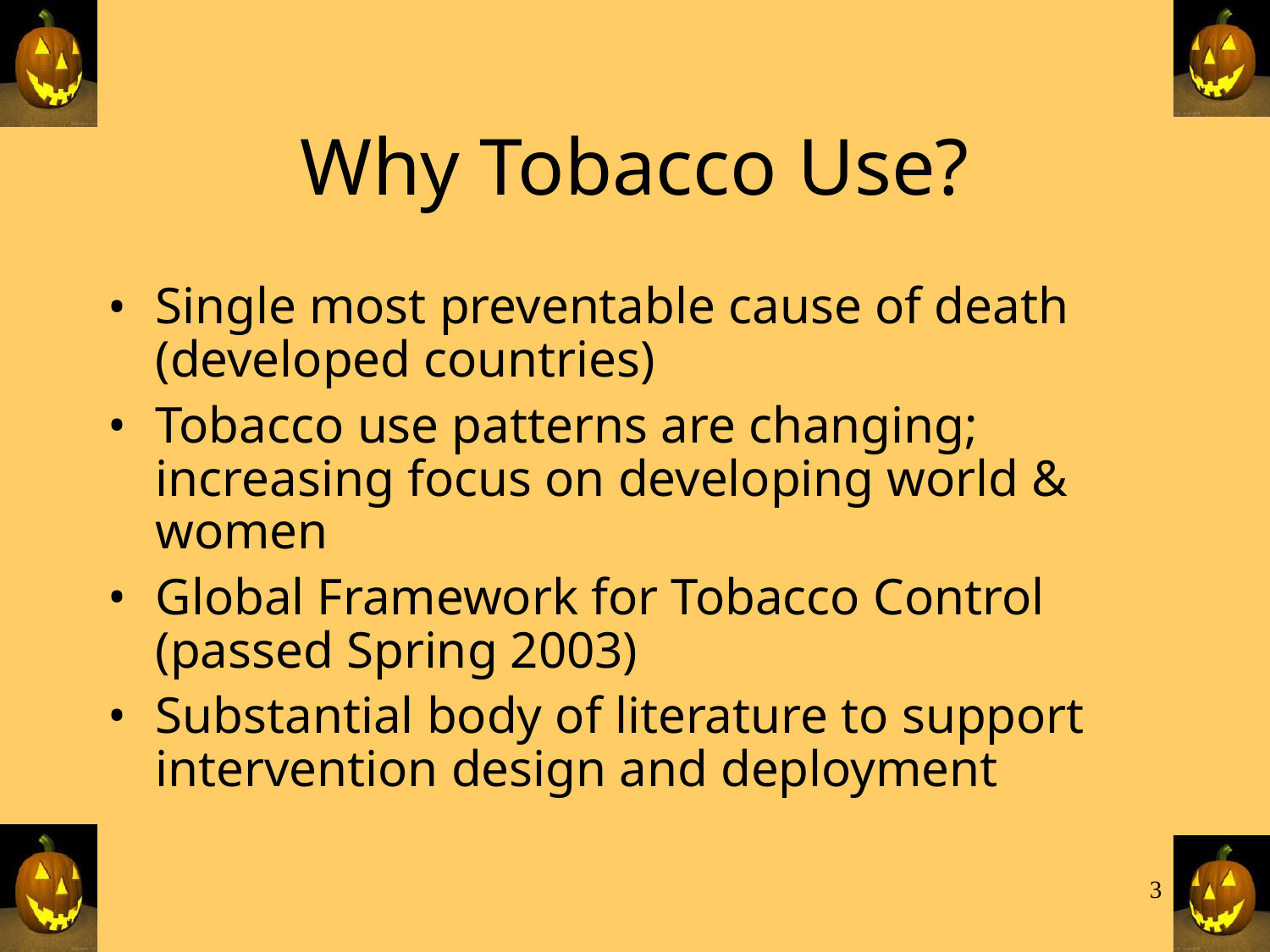

# Why Tobacco Use?
Single most preventable cause of death (developed countries)
Tobacco use patterns are changing; increasing focus on developing world & women
Global Framework for Tobacco Control (passed Spring 2003)
Substantial body of literature to support intervention design and deployment
3

## Slide 4
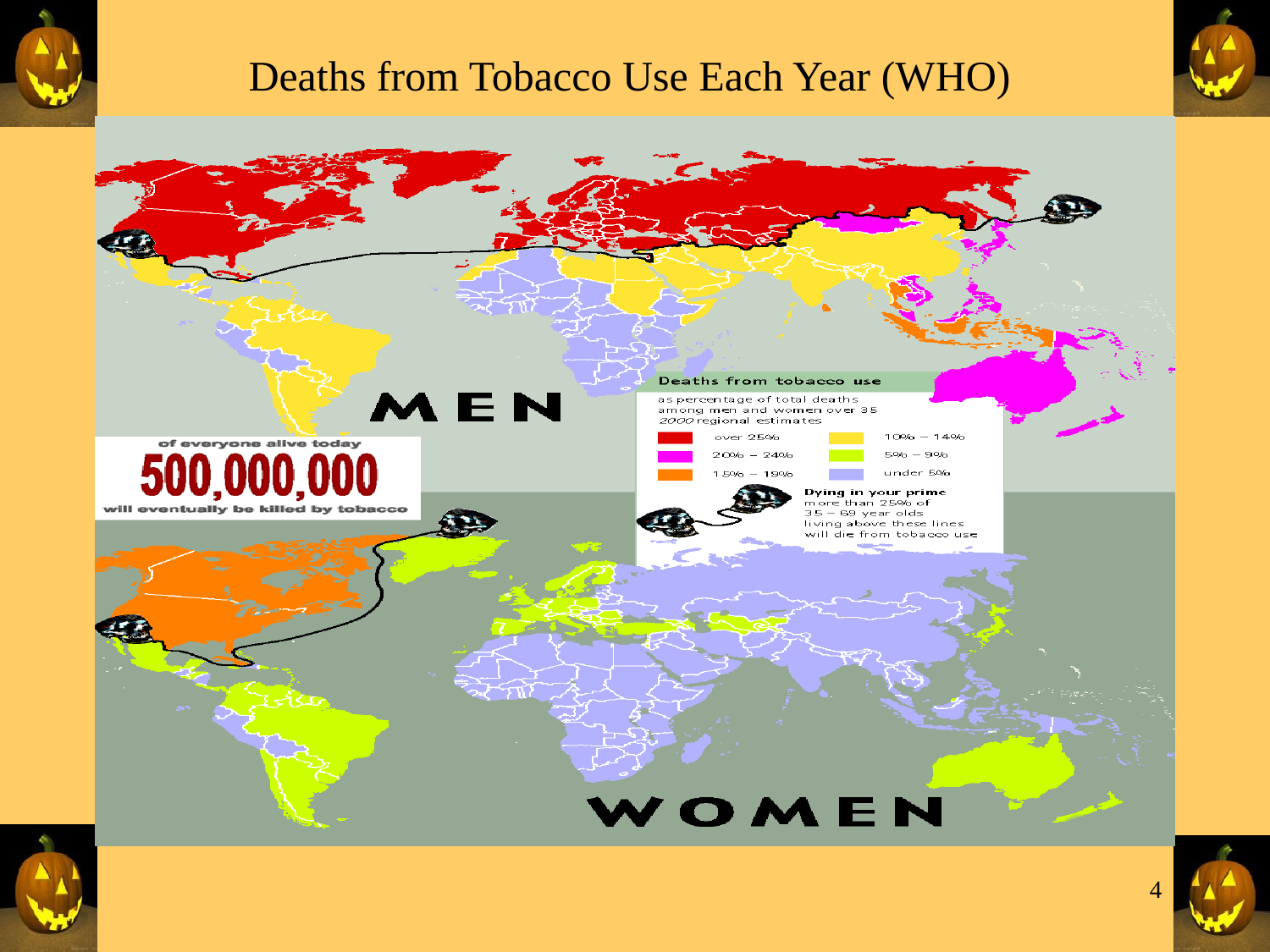

Deaths from Tobacco Use Each Year (WHO)
4

## Slide 5
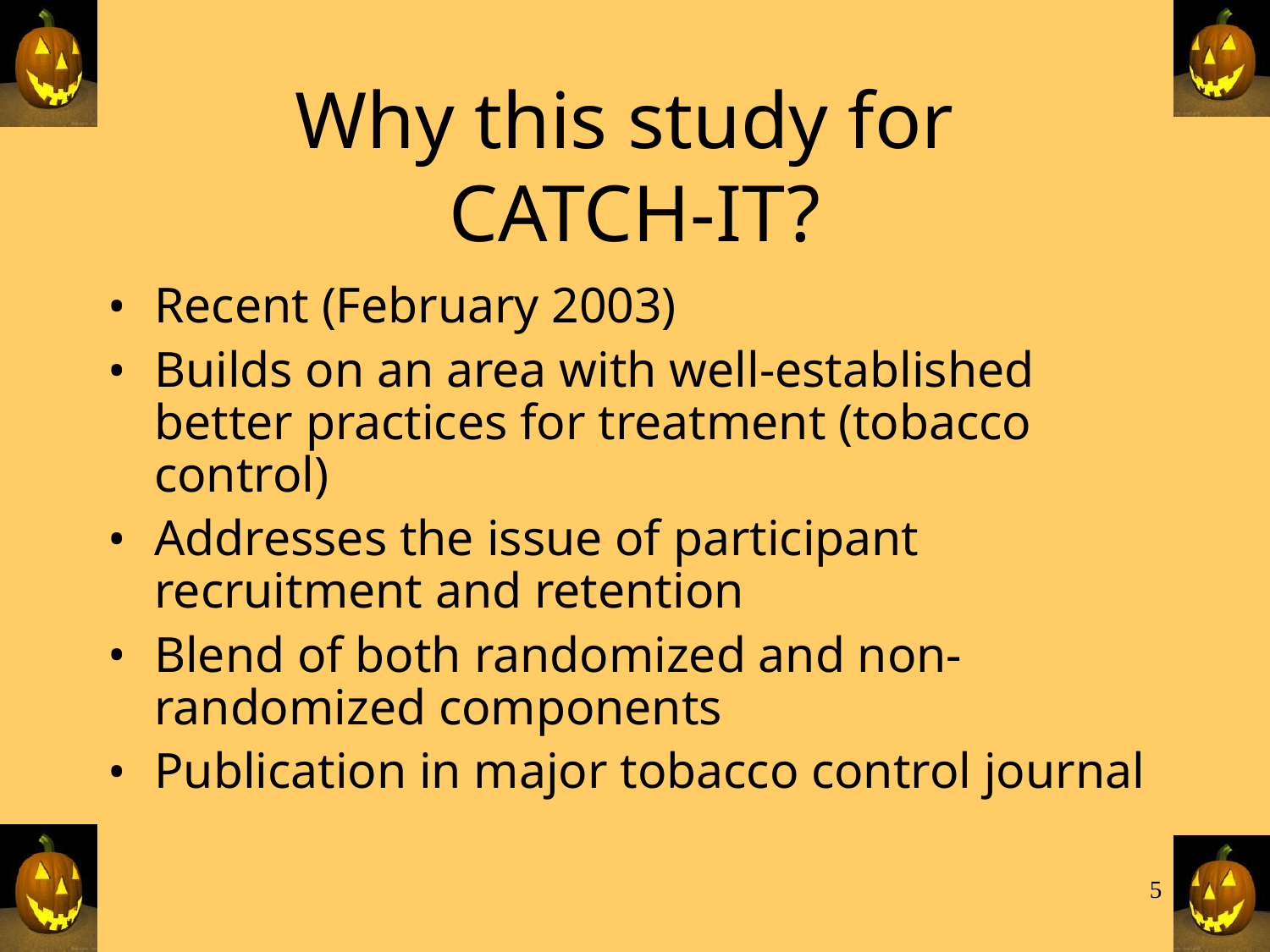

# Why this study for CATCH-IT?
Recent (February 2003)
Builds on an area with well-established better practices for treatment (tobacco control)
Addresses the issue of participant recruitment and retention
Blend of both randomized and non-randomized components
Publication in major tobacco control journal
5

## Slide 6
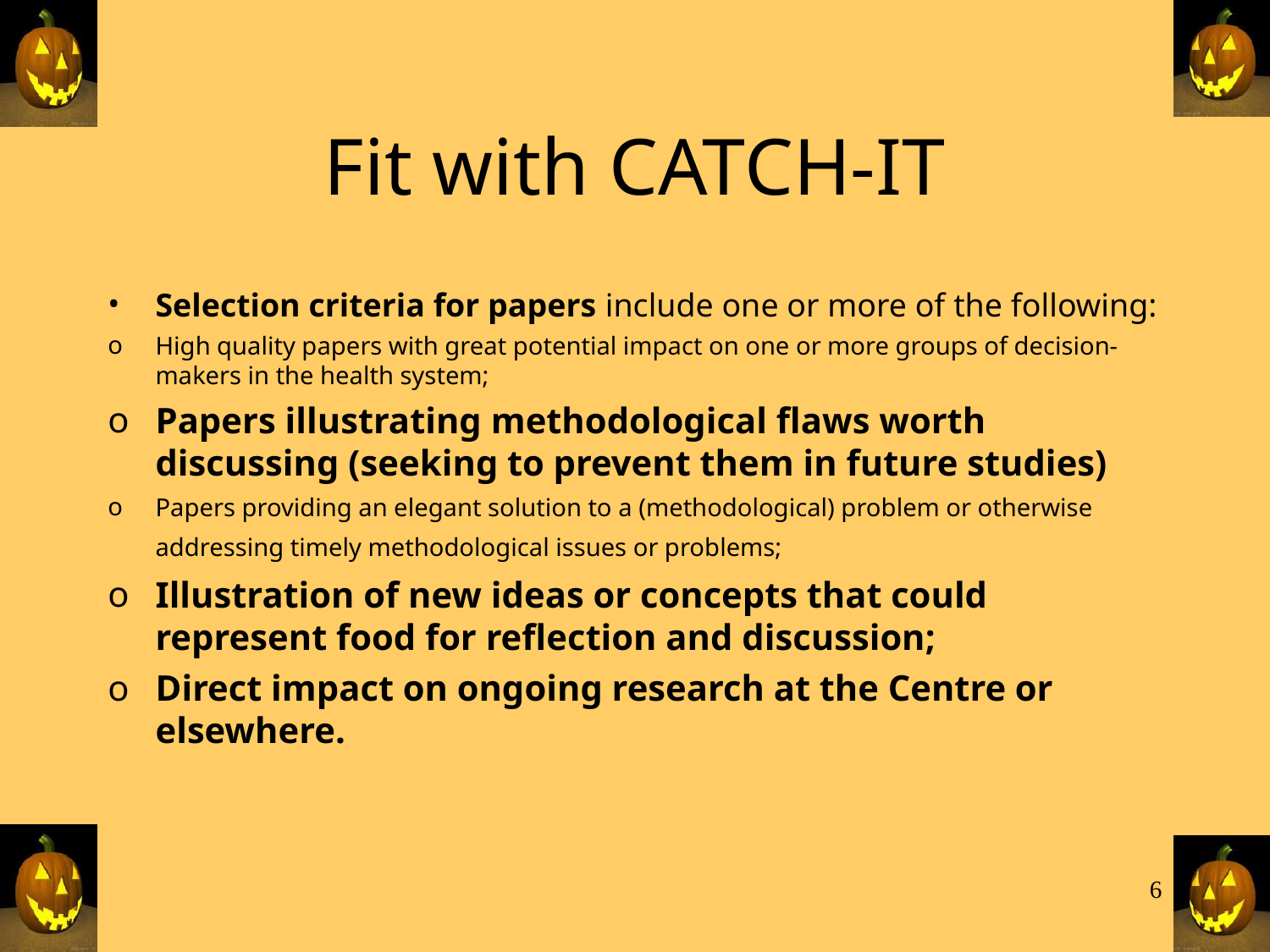

# Fit with CATCH-IT
Selection criteria for papers include one or more of the following:
High quality papers with great potential impact on one or more groups of decision-makers in the health system;
Papers illustrating methodological flaws worth discussing (seeking to prevent them in future studies)
Papers providing an elegant solution to a (methodological) problem or otherwise addressing timely methodological issues or problems;
Illustration of new ideas or concepts that could represent food for reflection and discussion;
Direct impact on ongoing research at the Centre or elsewhere.
6

## Slide 7
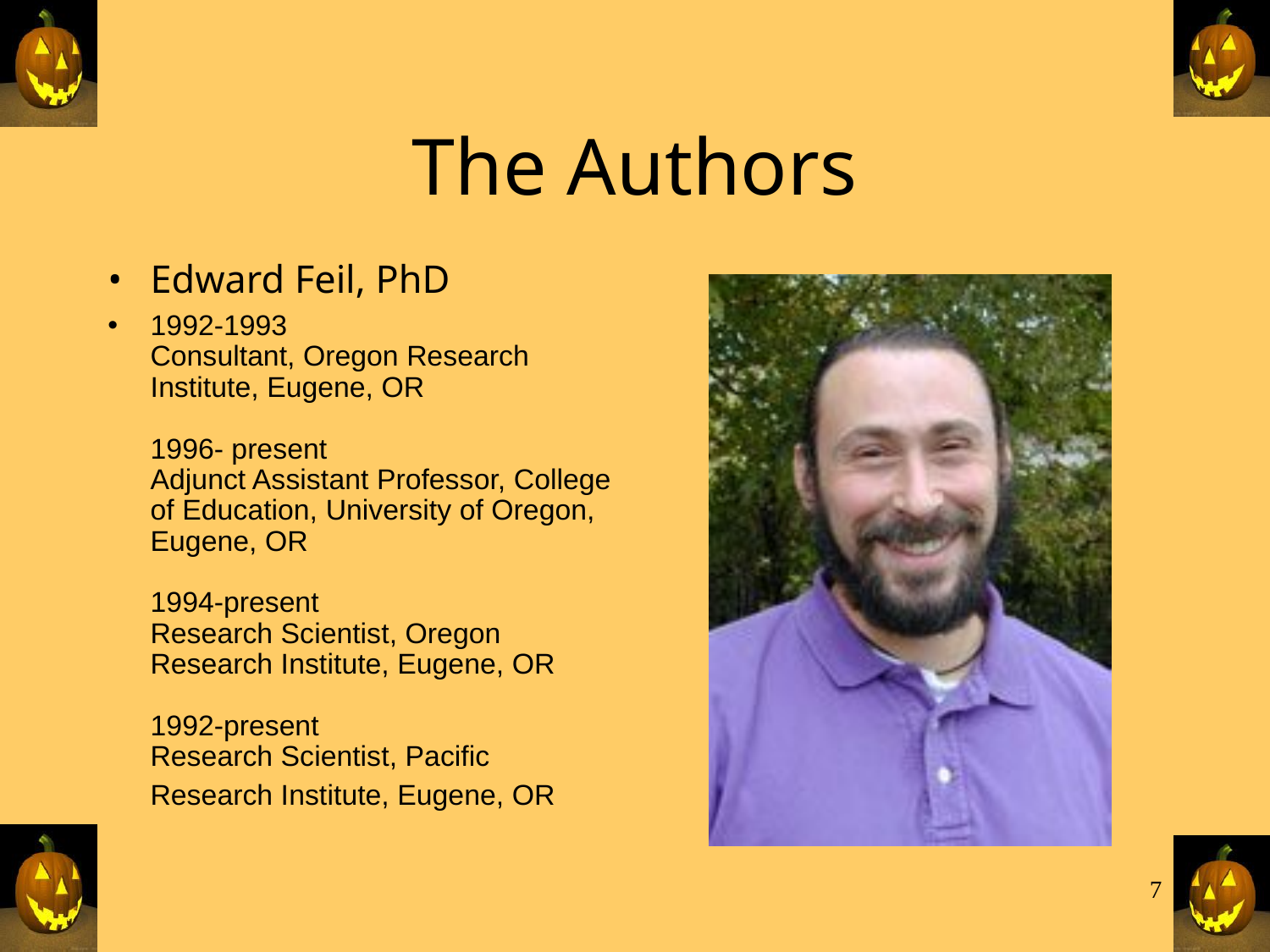

# The Authors
Edward Feil, PhD
1992-1993
Consultant, Oregon Research Institute, Eugene, OR
1996- present
Adjunct Assistant Professor, College of Education, University of Oregon, Eugene, OR
1994-present
Research Scientist, Oregon Research Institute, Eugene, OR
1992-present
Research Scientist, Pacific Research Institute, Eugene, OR
7

## Slide 8
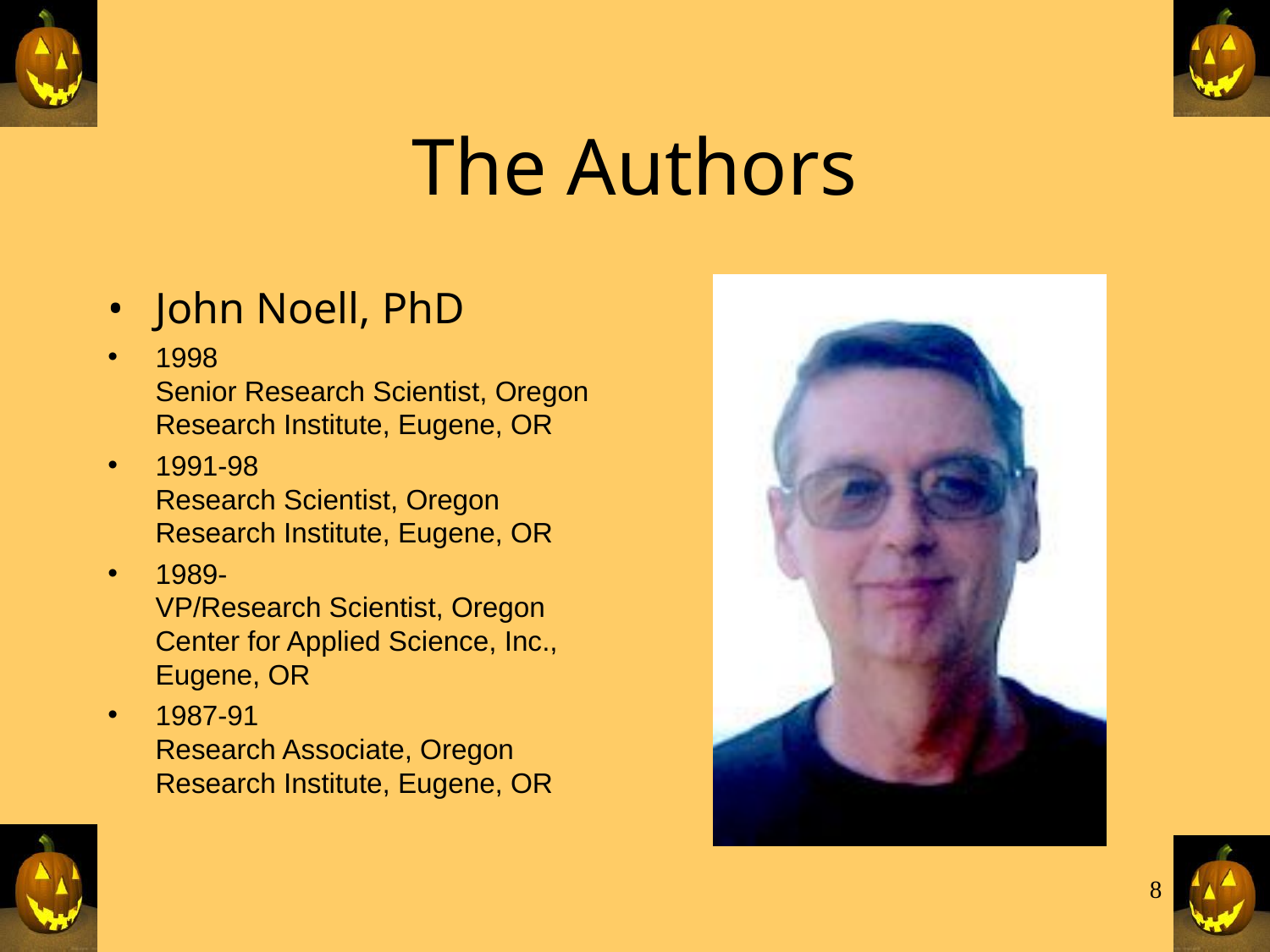

1998
Senior Research Scientist, Oregon Research Institute, Eugene, OR
1991-98
Research Scientist, Oregon Research Institute, Eugene, OR
1989-
VP/Research Scientist, Oregon Center for Applied Science, Inc., Eugene, OR
1987-91
Research Associate, Oregon Research Institute, Eugene, OR
1983-87
Science Specialist/Instructional Video Designer, Engelmann-Becker Corporation, Eugene, OR
# The Authors
John Noell, PhD
1998
Senior Research Scientist, Oregon Research Institute, Eugene, OR
1991-98
Research Scientist, Oregon Research Institute, Eugene, OR
1989-
VP/Research Scientist, Oregon Center for Applied Science, Inc., Eugene, OR
1987-91
Research Associate, Oregon Research Institute, Eugene, OR
8

## Slide 9
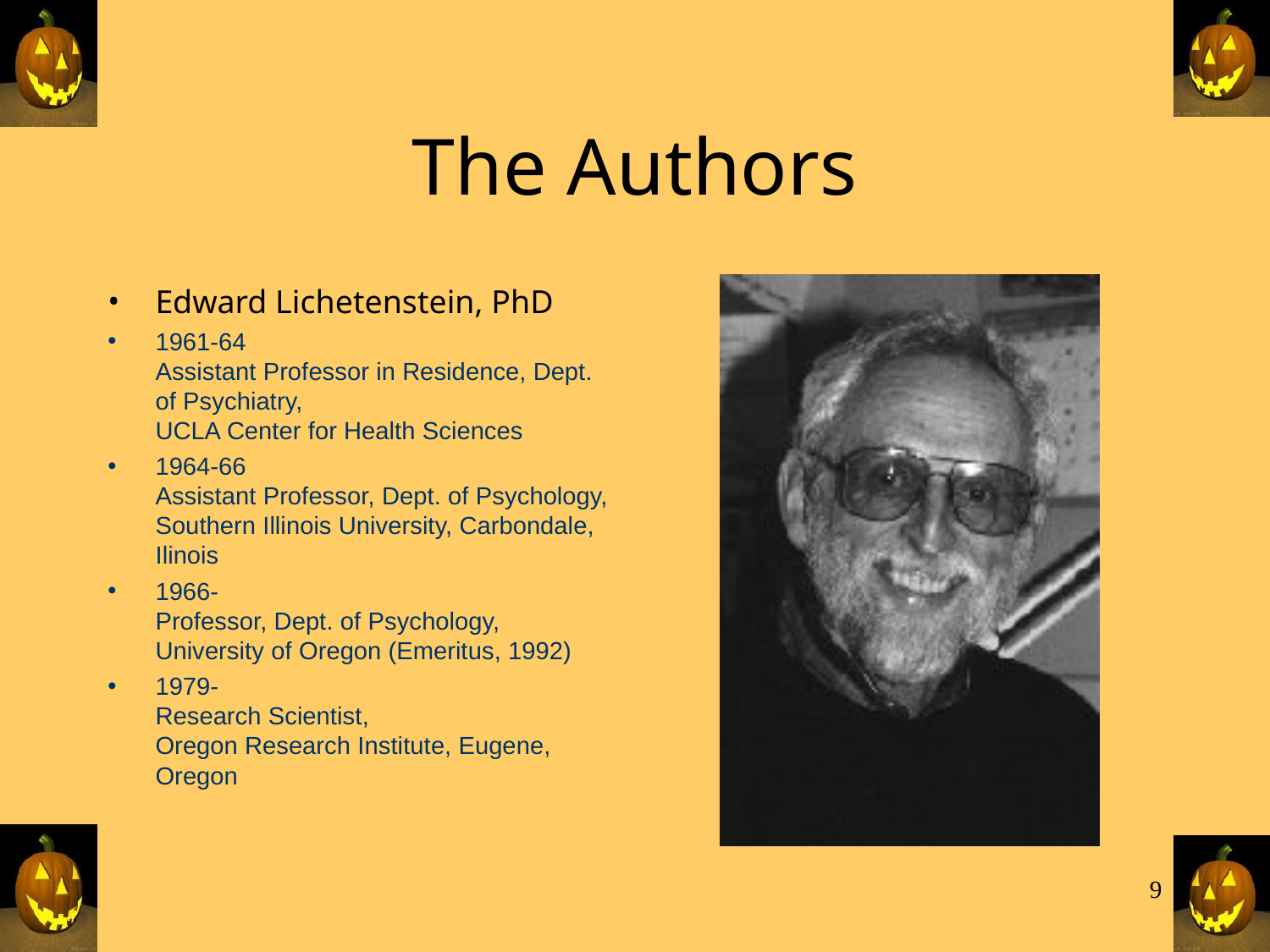

# The Authors
Edward Lichetenstein, PhD
1961-64
Assistant Professor in Residence, Dept. of Psychiatry,
UCLA Center for Health Sciences
1964-66
Assistant Professor, Dept. of Psychology,
Southern Illinois University, Carbondale, Ilinois
1966-
Professor, Dept. of Psychology,
University of Oregon (Emeritus, 1992)
1979-
Research Scientist,
Oregon Research Institute, Eugene, Oregon
9

## Slide 10
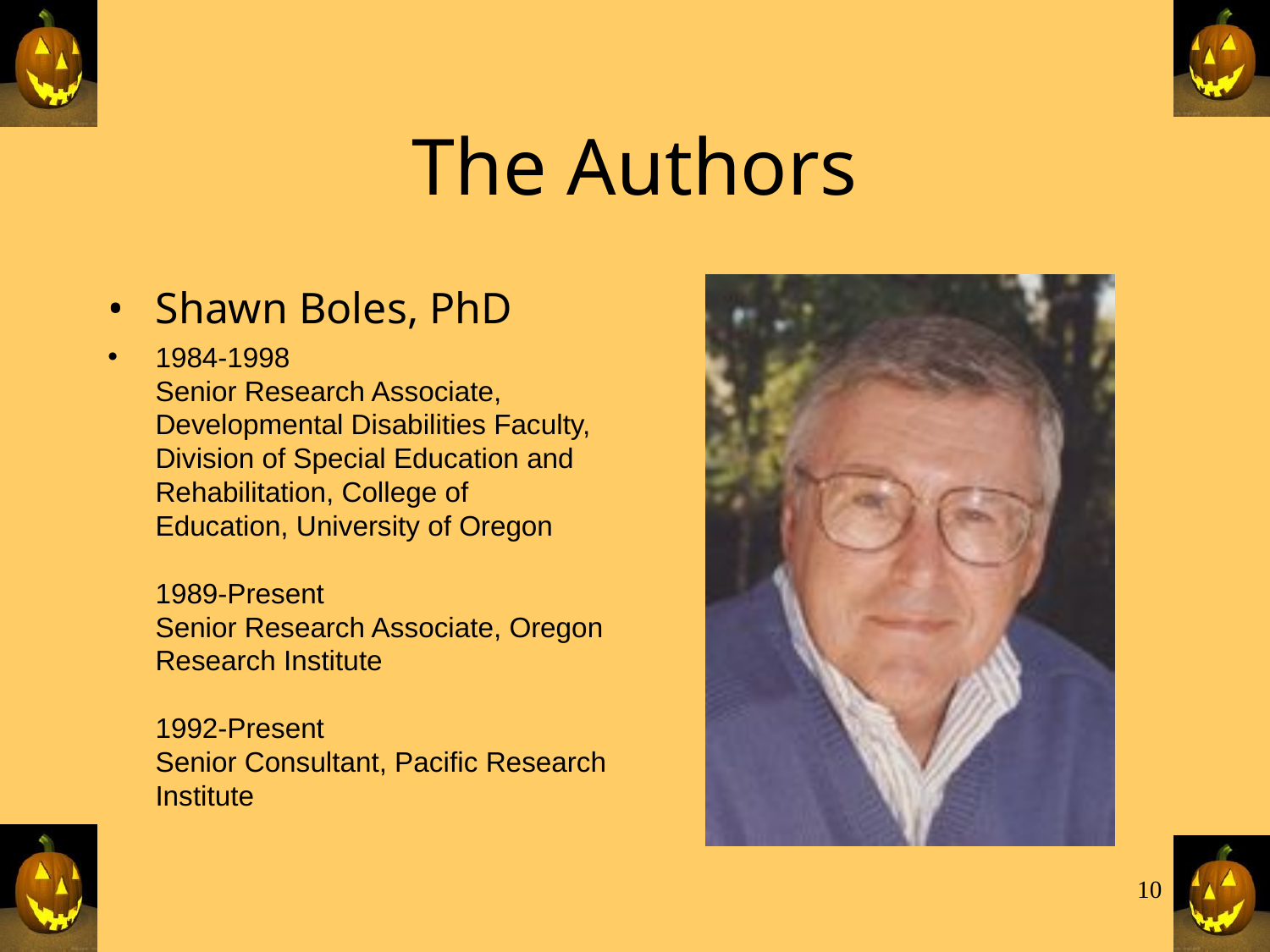

# The Authors
Shawn Boles, PhD
1984-1998
Senior Research Associate, Developmental Disabilities Faculty, Division of Special Education and Rehabilitation, College of
Education, University of Oregon
1989-Present
Senior Research Associate, Oregon Research Institute
1992-Present
Senior Consultant, Pacific Research Institute
10

## Slide 11
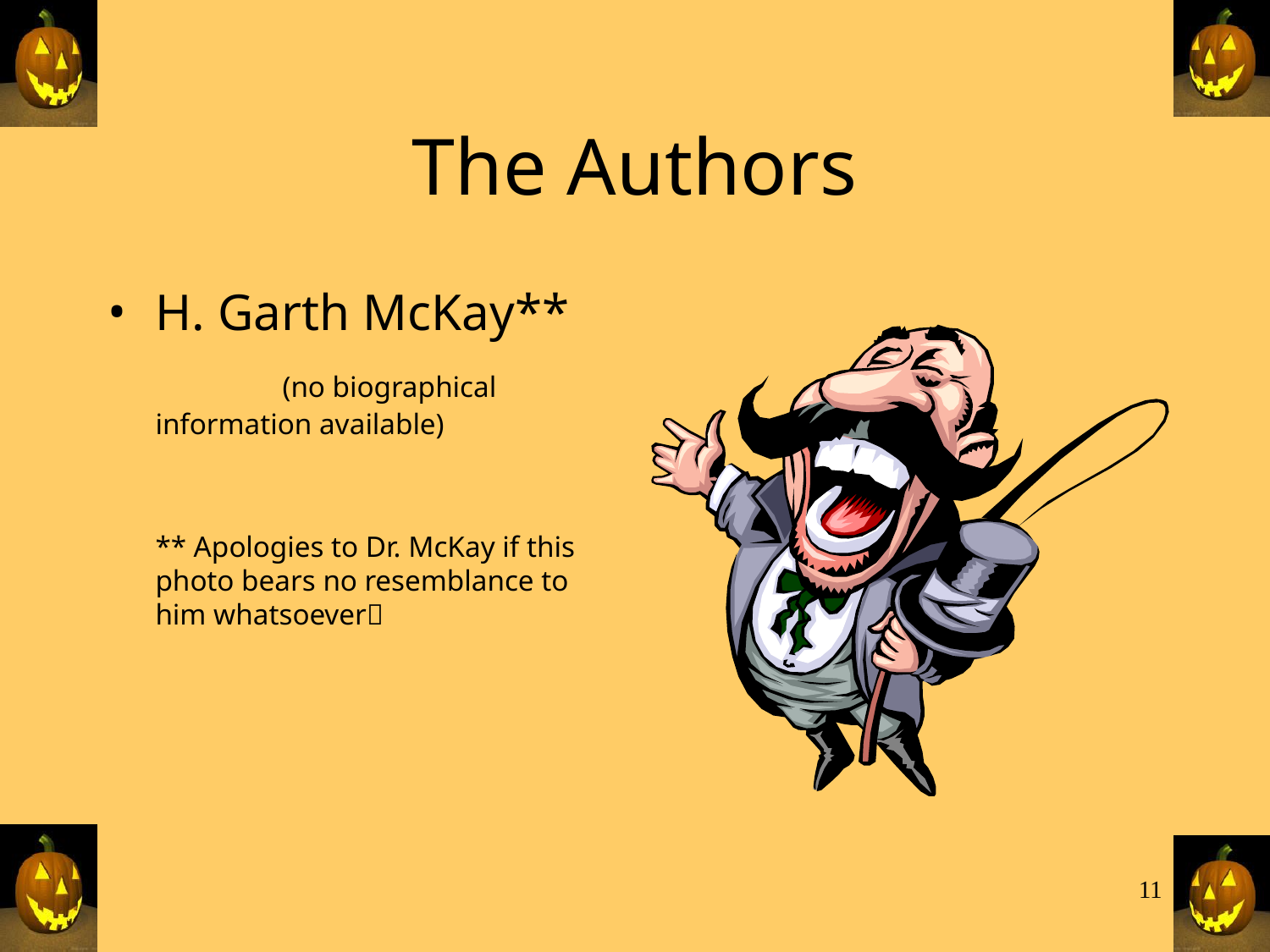

# The Authors
H. Garth McKay**
	(no biographical information available)
** Apologies to Dr. McKay if this photo bears no resemblance to him whatsoever
11

## Slide 12
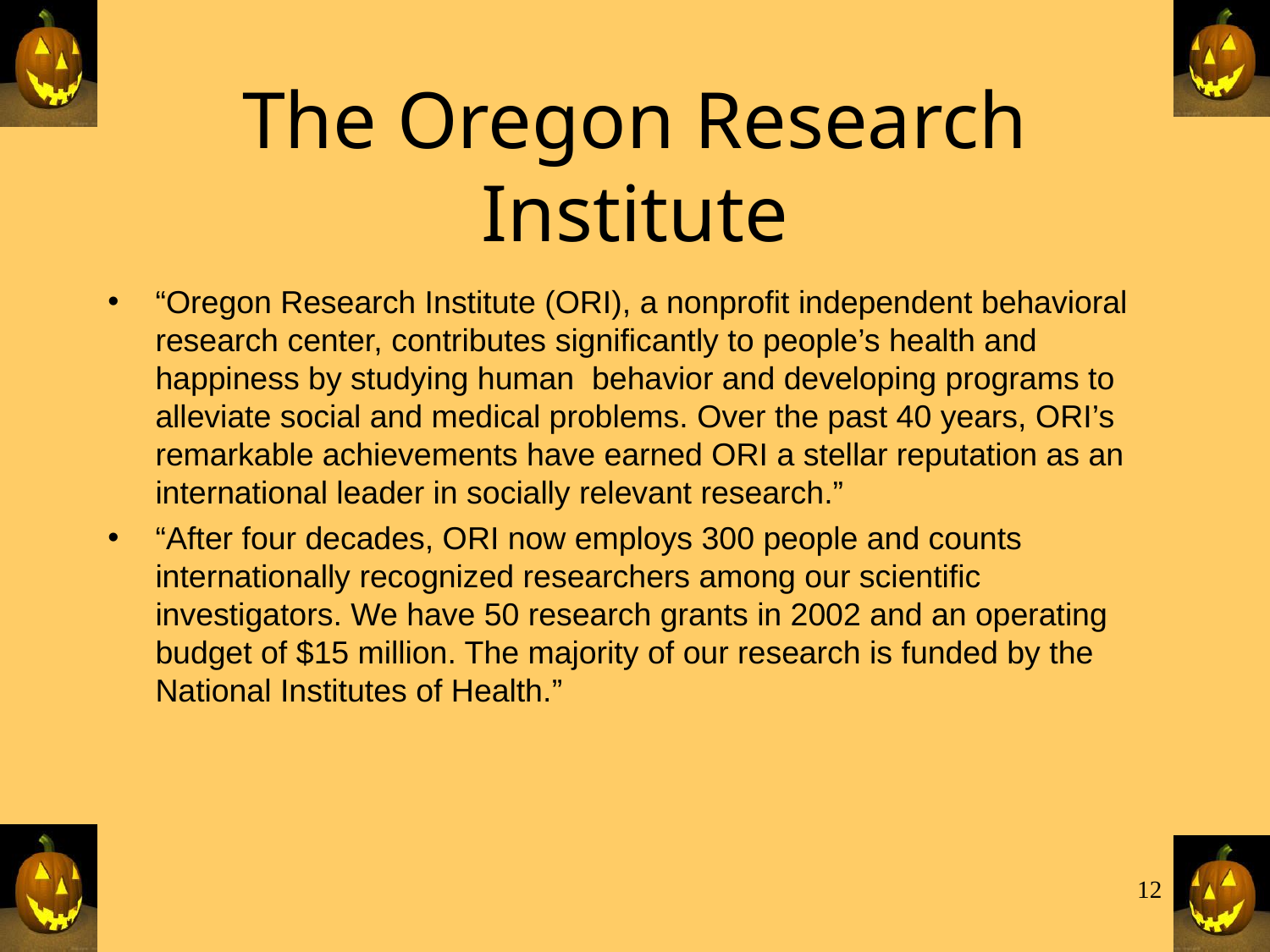

# The Oregon Research Institute
“Oregon Research Institute (ORI), a nonprofit independent behavioral research center, contributes significantly to people’s health and happiness by studying human behavior and developing programs to alleviate social and medical problems. Over the past 40 years, ORI’s remarkable achievements have earned ORI a stellar reputation as an international leader in socially relevant research.”
“After four decades, ORI now employs 300 people and counts internationally recognized researchers among our scientific investigators. We have 50 research grants in 2002 and an operating budget of $15 million. The majority of our research is funded by the National Institutes of Health.”
12

## Slide 13
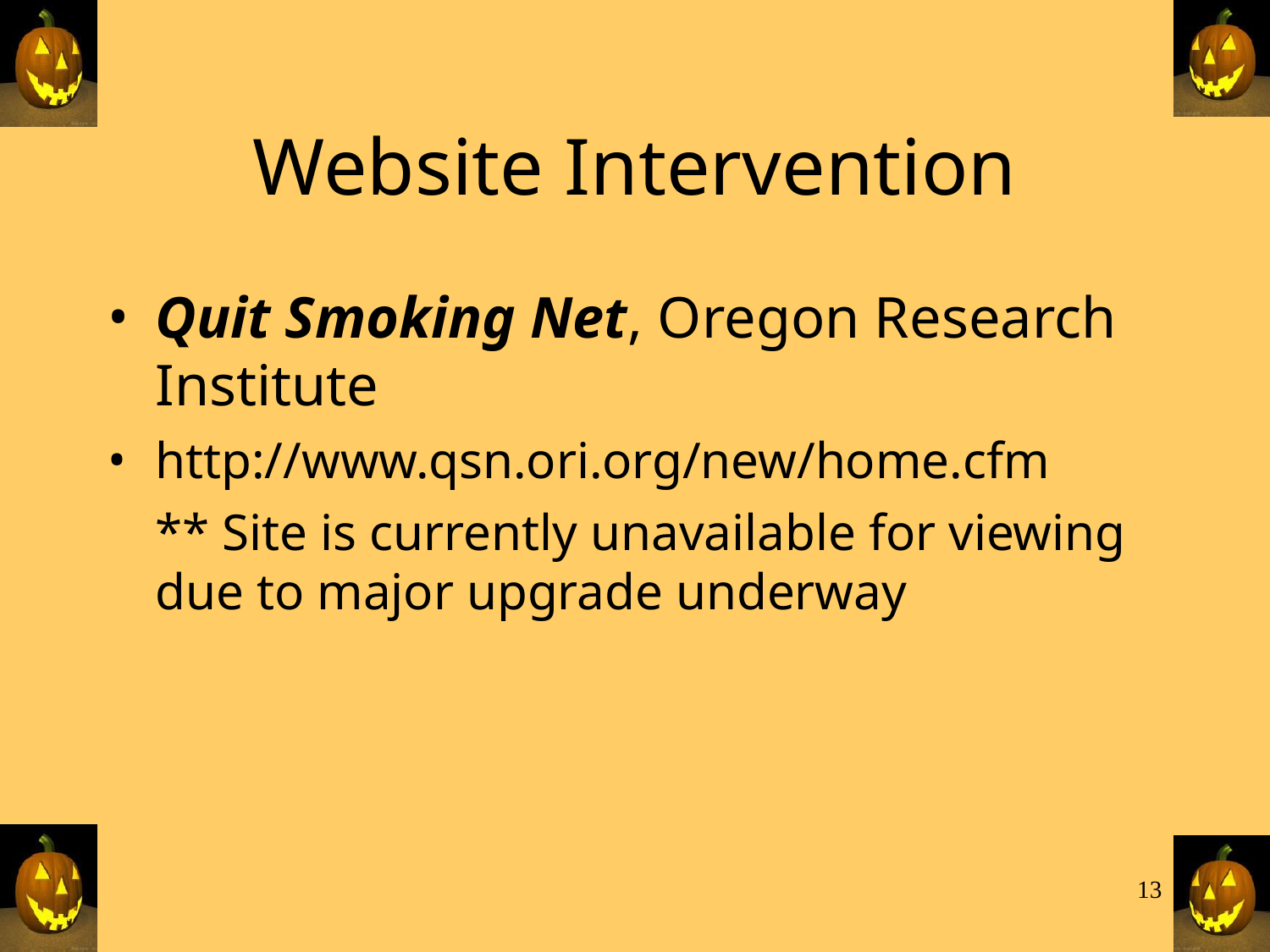

# Website Intervention
Quit Smoking Net, Oregon Research Institute
http://www.qsn.ori.org/new/home.cfm
** Site is currently unavailable for viewing due to major upgrade underway
13

## Slide 14
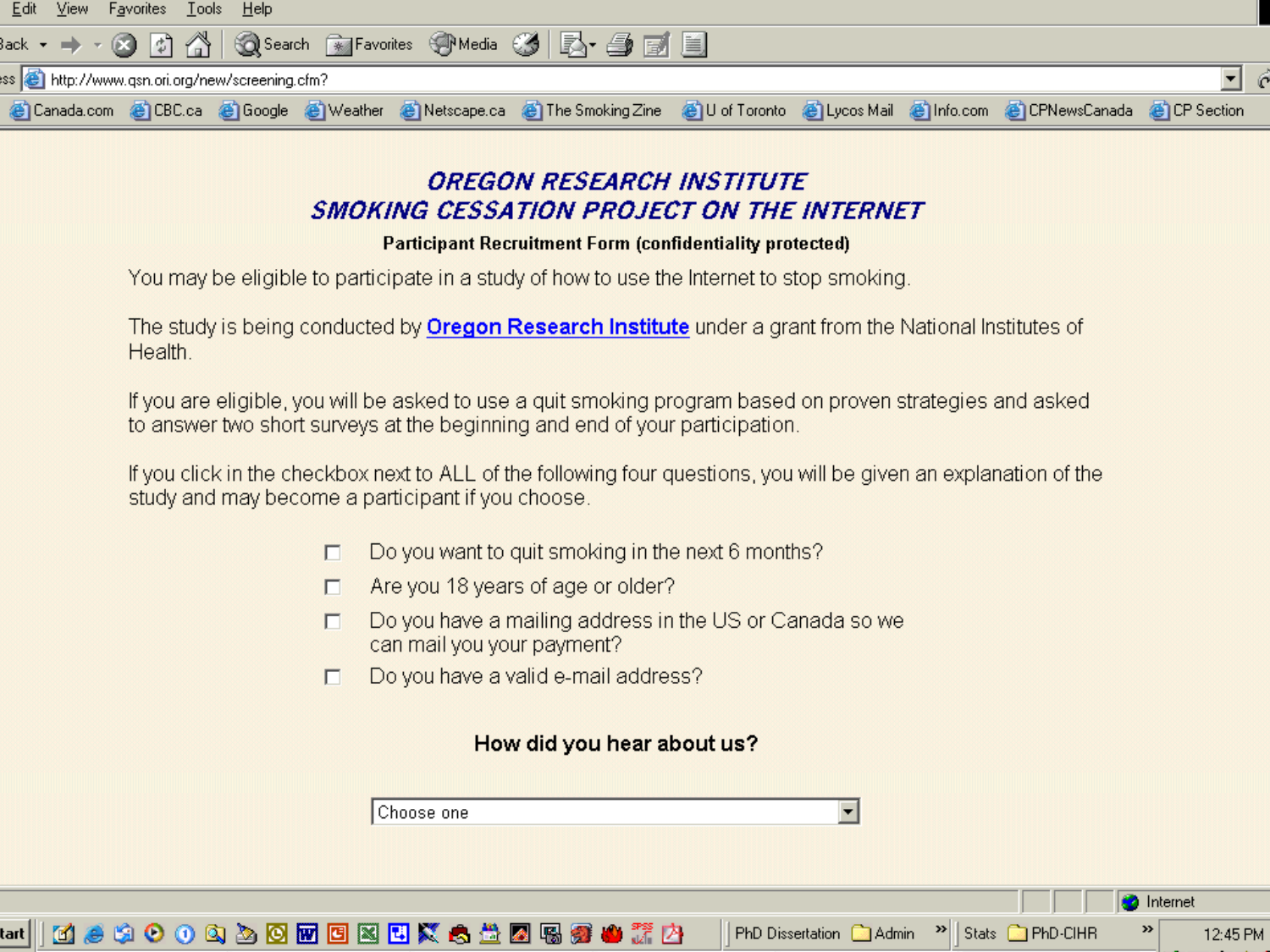

14

## Slide 15
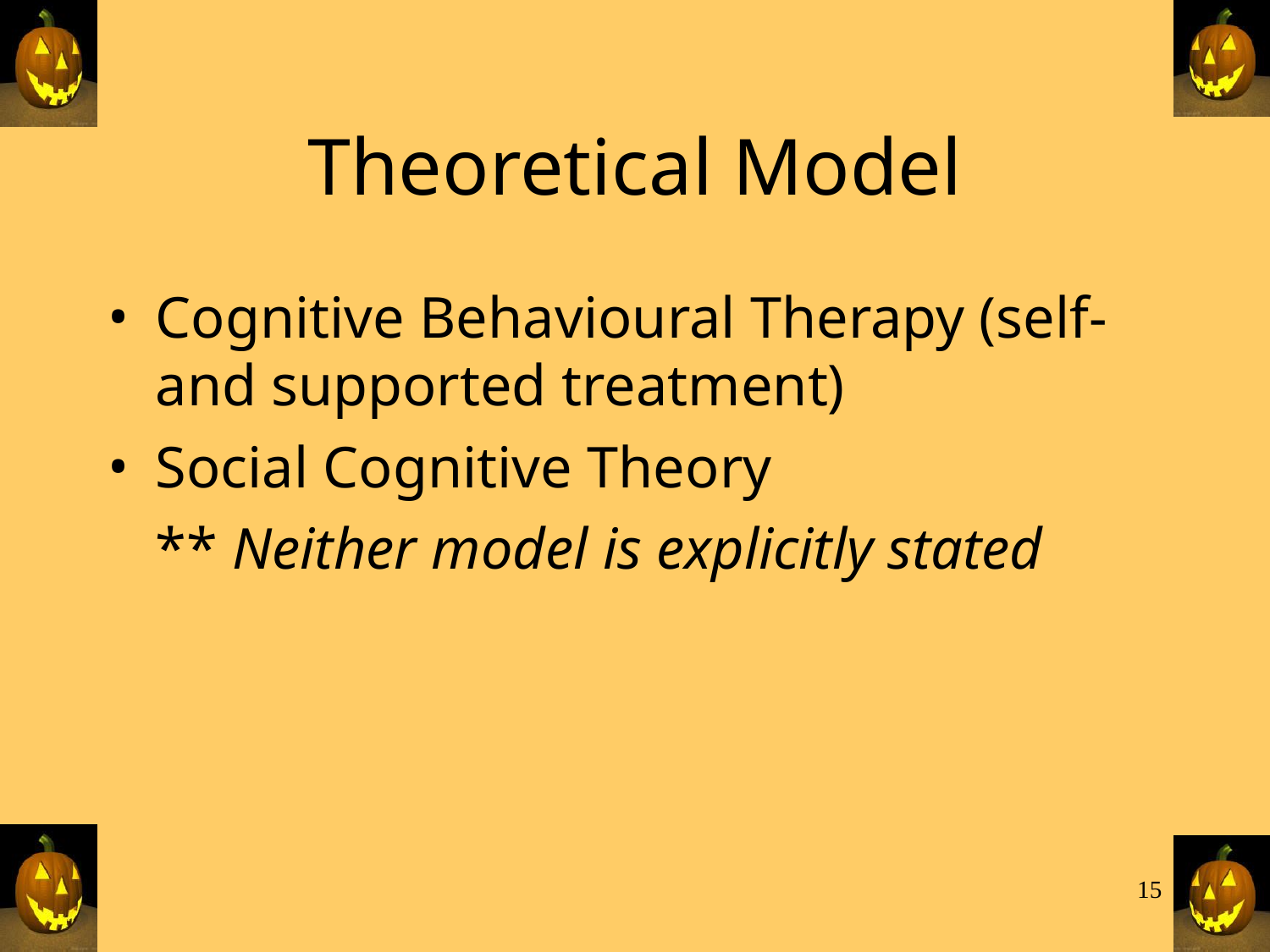

# Theoretical Model
Cognitive Behavioural Therapy (self- and supported treatment)
Social Cognitive Theory
** Neither model is explicitly stated
15

## Slide 16
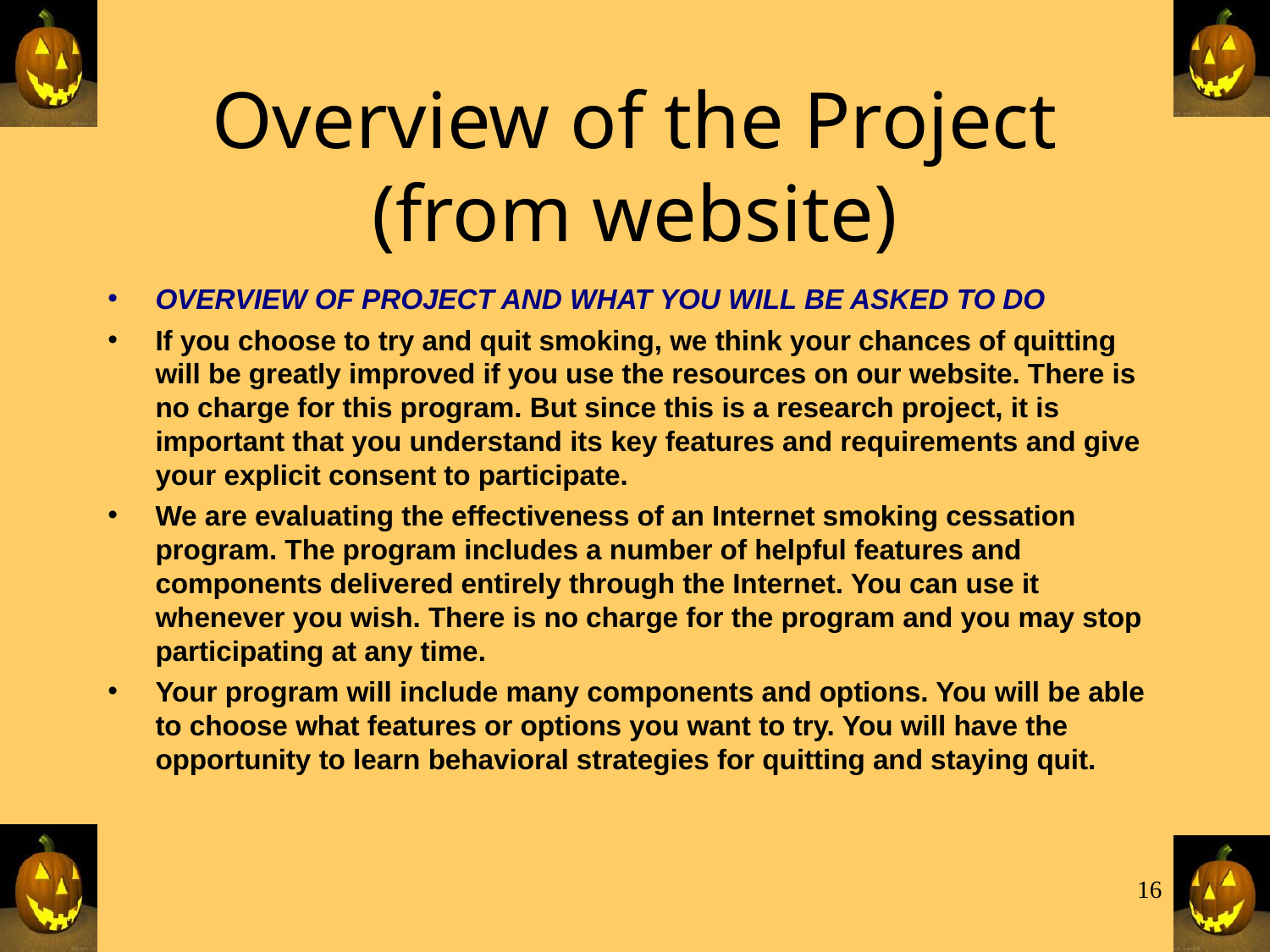

# Overview of the Project (from website)
OVERVIEW OF PROJECT AND WHAT YOU WILL BE ASKED TO DO
If you choose to try and quit smoking, we think your chances of quitting will be greatly improved if you use the resources on our website. There is no charge for this program. But since this is a research project, it is important that you understand its key features and requirements and give your explicit consent to participate.
We are evaluating the effectiveness of an Internet smoking cessation program. The program includes a number of helpful features and components delivered entirely through the Internet. You can use it whenever you wish. There is no charge for the program and you may stop participating at any time.
Your program will include many components and options. You will be able to choose what features or options you want to try. You will have the opportunity to learn behavioral strategies for quitting and staying quit.
16

## Slide 17
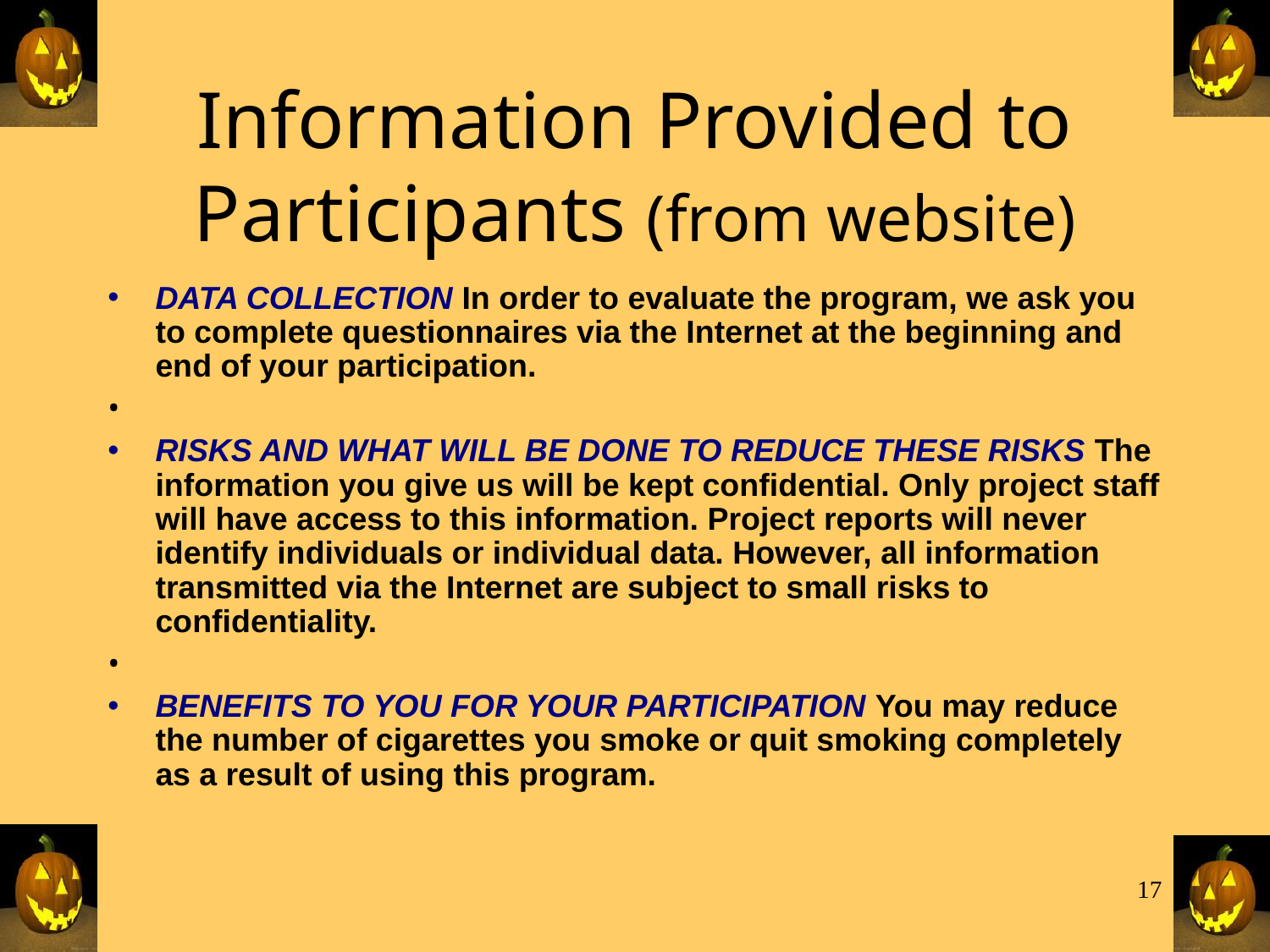

# Information Provided to Participants (from website)
DATA COLLECTION In order to evaluate the program, we ask you to complete questionnaires via the Internet at the beginning and end of your participation.
RISKS AND WHAT WILL BE DONE TO REDUCE THESE RISKS The information you give us will be kept confidential. Only project staff will have access to this information. Project reports will never identify individuals or individual data. However, all information transmitted via the Internet are subject to small risks to confidentiality.
BENEFITS TO YOU FOR YOUR PARTICIPATION You may reduce the number of cigarettes you smoke or quit smoking completely as a result of using this program.
17

## Slide 18
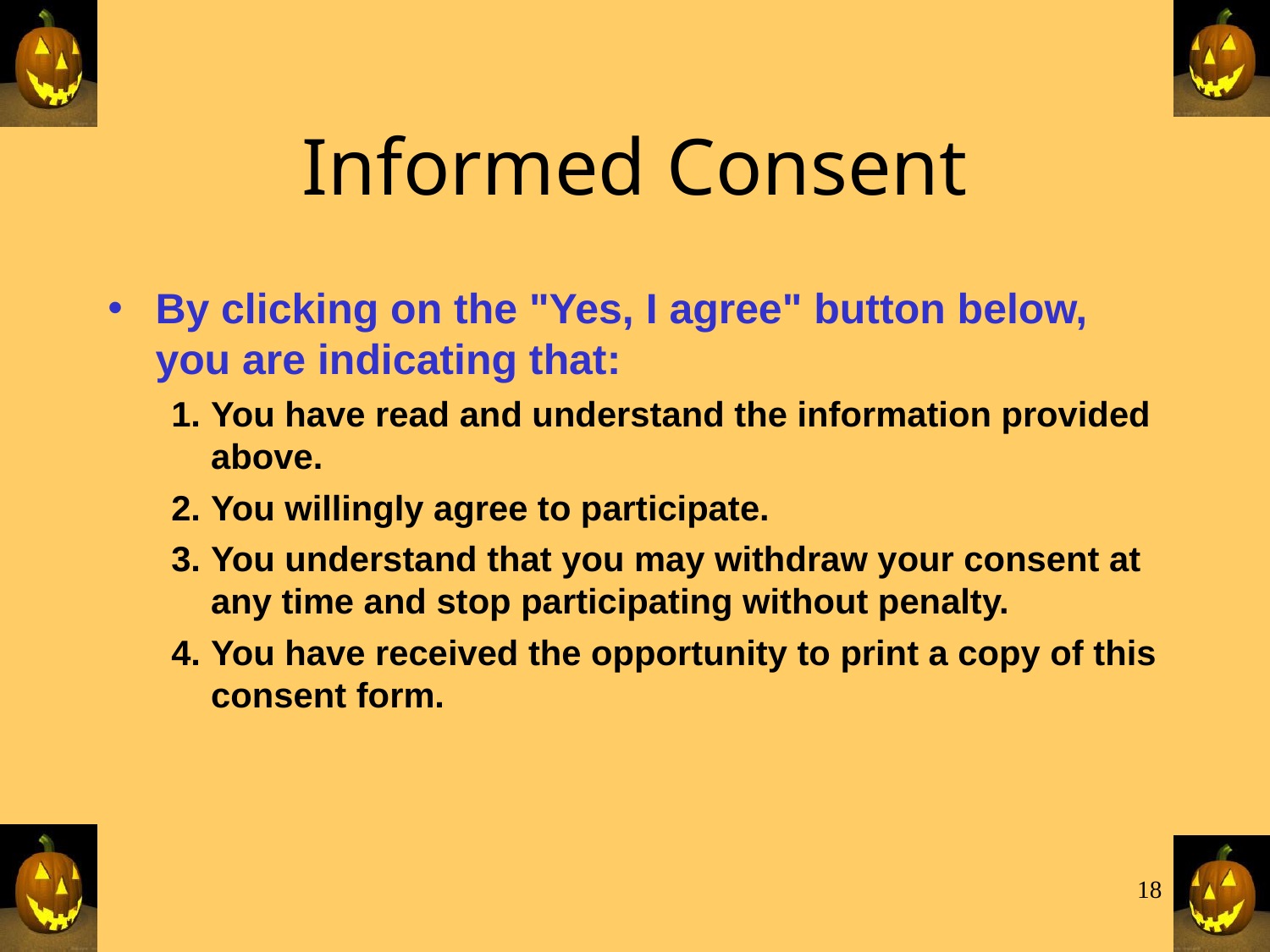

# Informed Consent
By clicking on the "Yes, I agree" button below, you are indicating that:
You have read and understand the information provided above.
You willingly agree to participate.
You understand that you may withdraw your consent at any time and stop participating without penalty.
You have received the opportunity to print a copy of this consent form.
18

## Slide 19
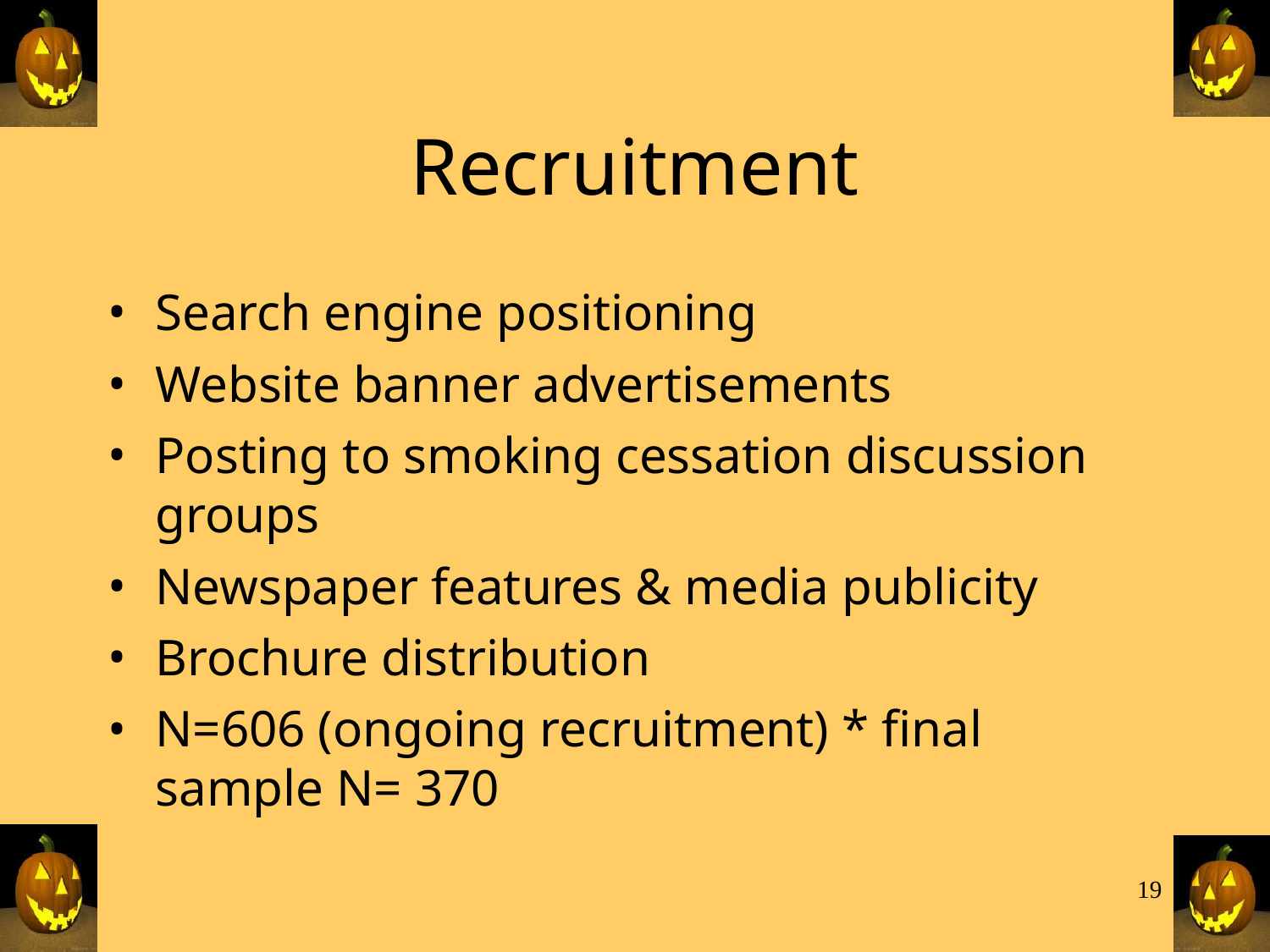

# Recruitment
Search engine positioning
Website banner advertisements
Posting to smoking cessation discussion groups
Newspaper features & media publicity
Brochure distribution
N=606 (ongoing recruitment) * final sample N= 370
19

## Slide 20
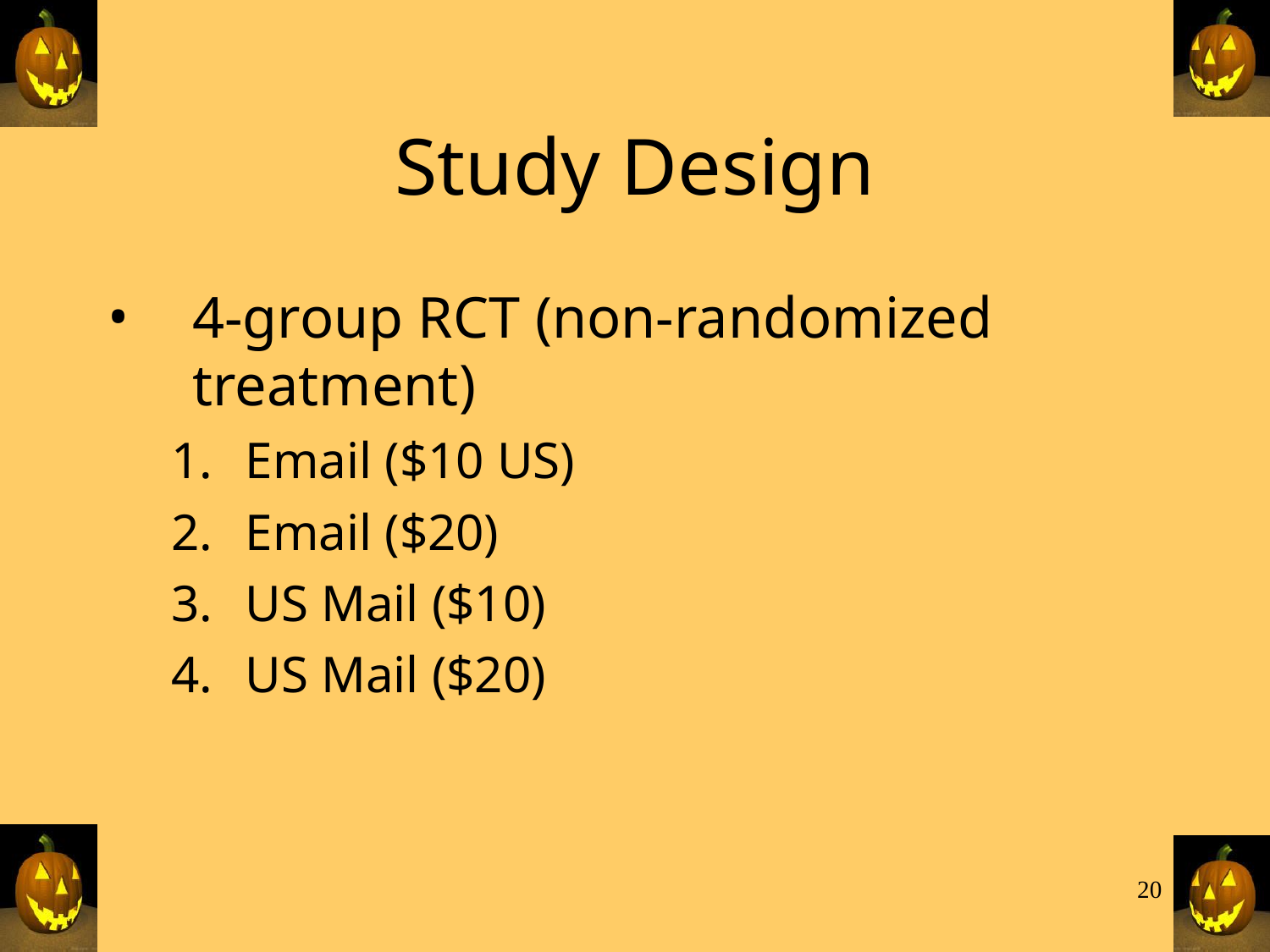

# Study Design
4-group RCT (non-randomized treatment)
Email ($10 US)
Email ($20)
US Mail ($10)
US Mail ($20)
20

## Slide 21
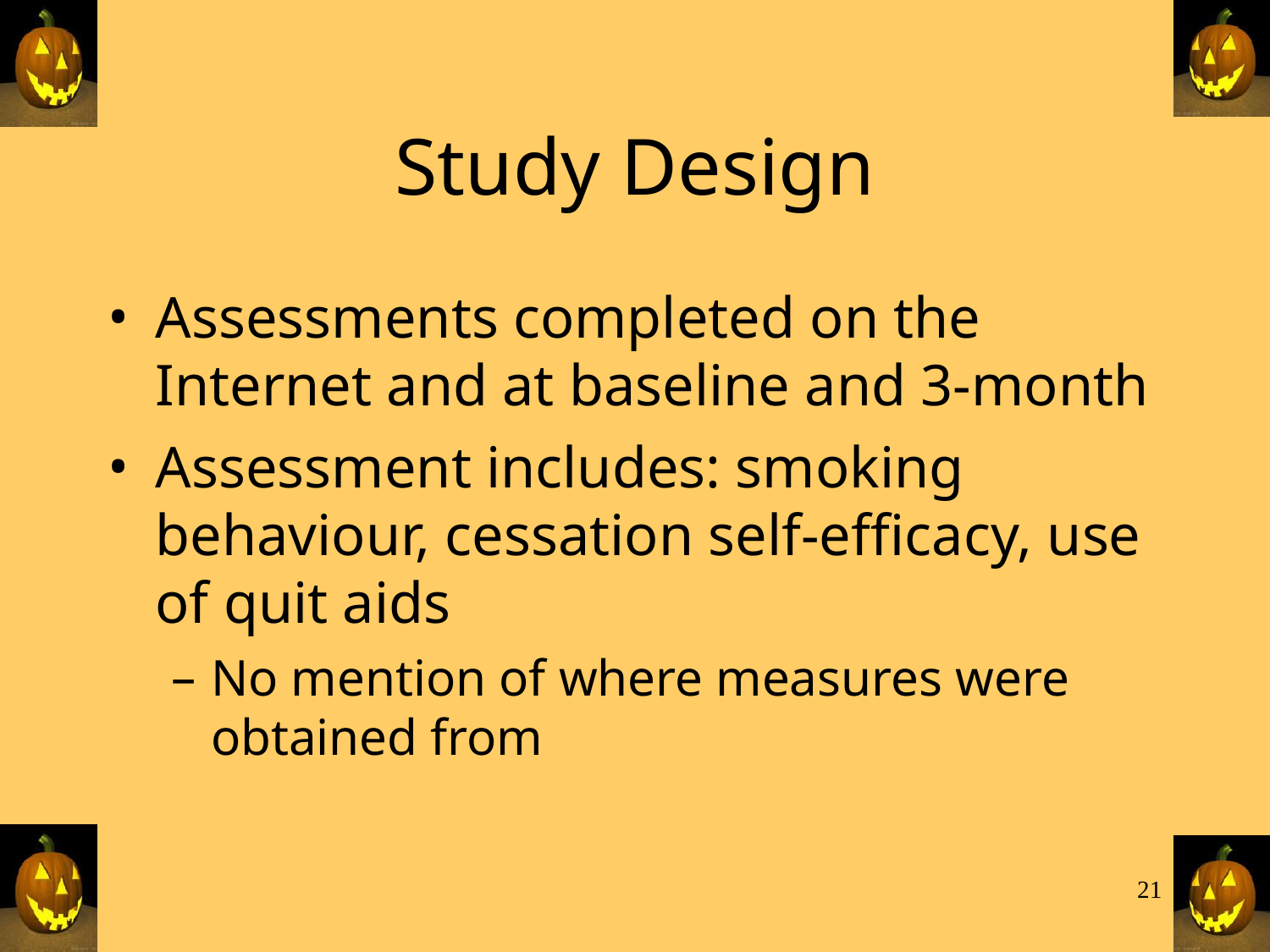

# Study Design
Assessments completed on the Internet and at baseline and 3-month
Assessment includes: smoking behaviour, cessation self-efficacy, use of quit aids
No mention of where measures were obtained from
21

## Slide 22
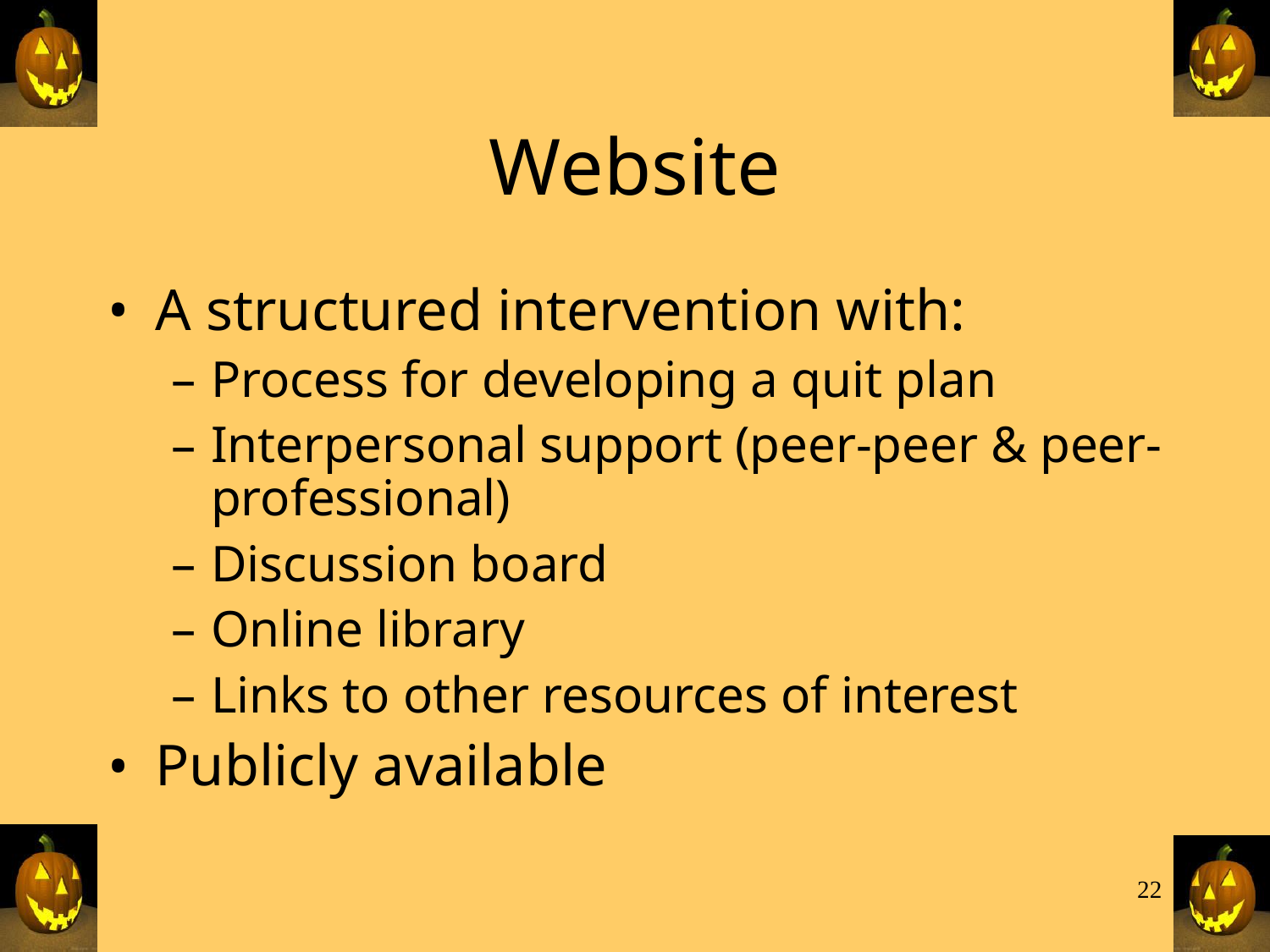

# Website
A structured intervention with:
Process for developing a quit plan
Interpersonal support (peer-peer & peer-professional)
Discussion board
Online library
Links to other resources of interest
Publicly available
22

## Slide 23
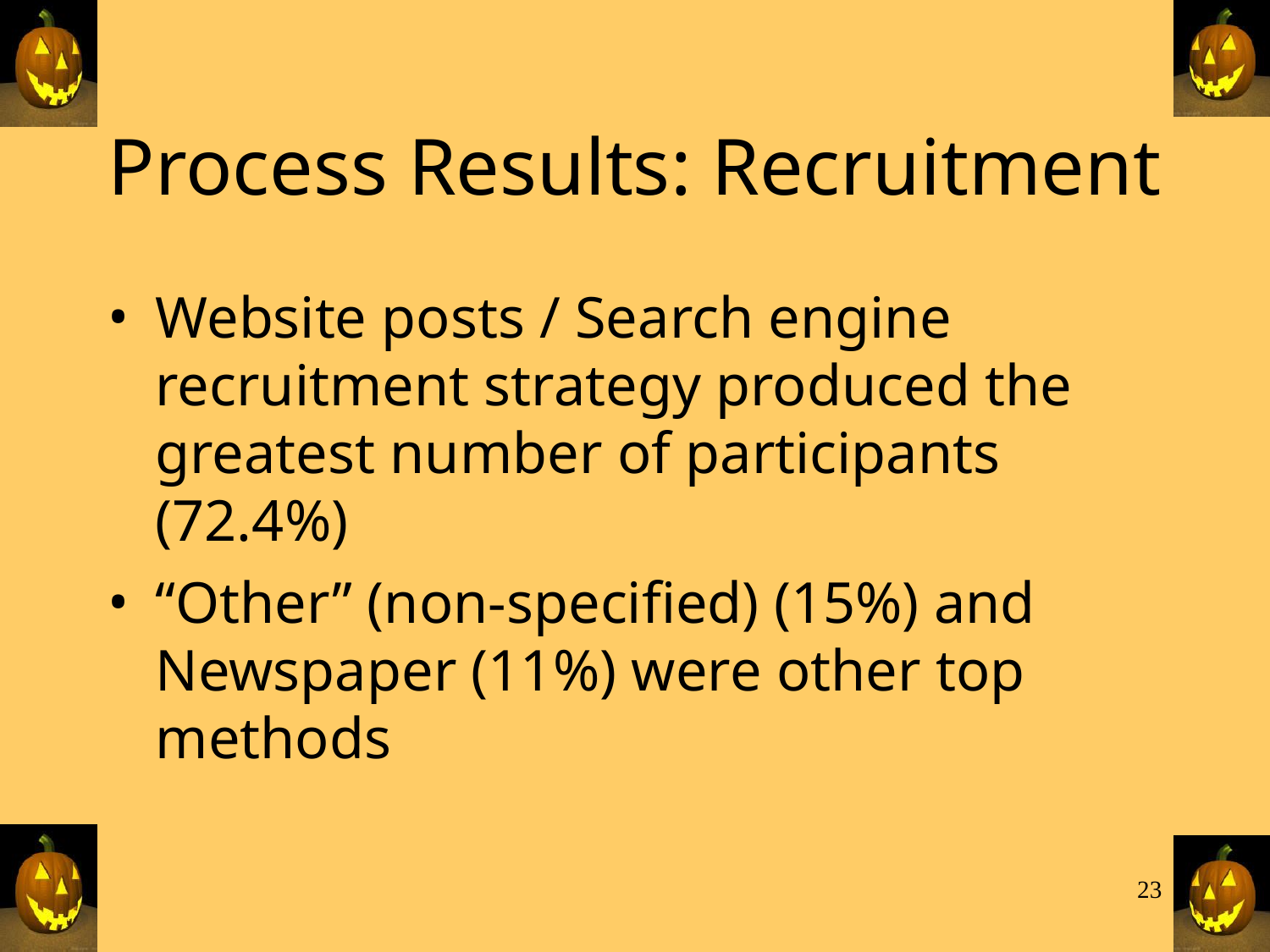

# Process Results: Recruitment
Website posts / Search engine recruitment strategy produced the greatest number of participants (72.4%)
“Other” (non-specified) (15%) and Newspaper (11%) were other top methods
23

## Slide 24
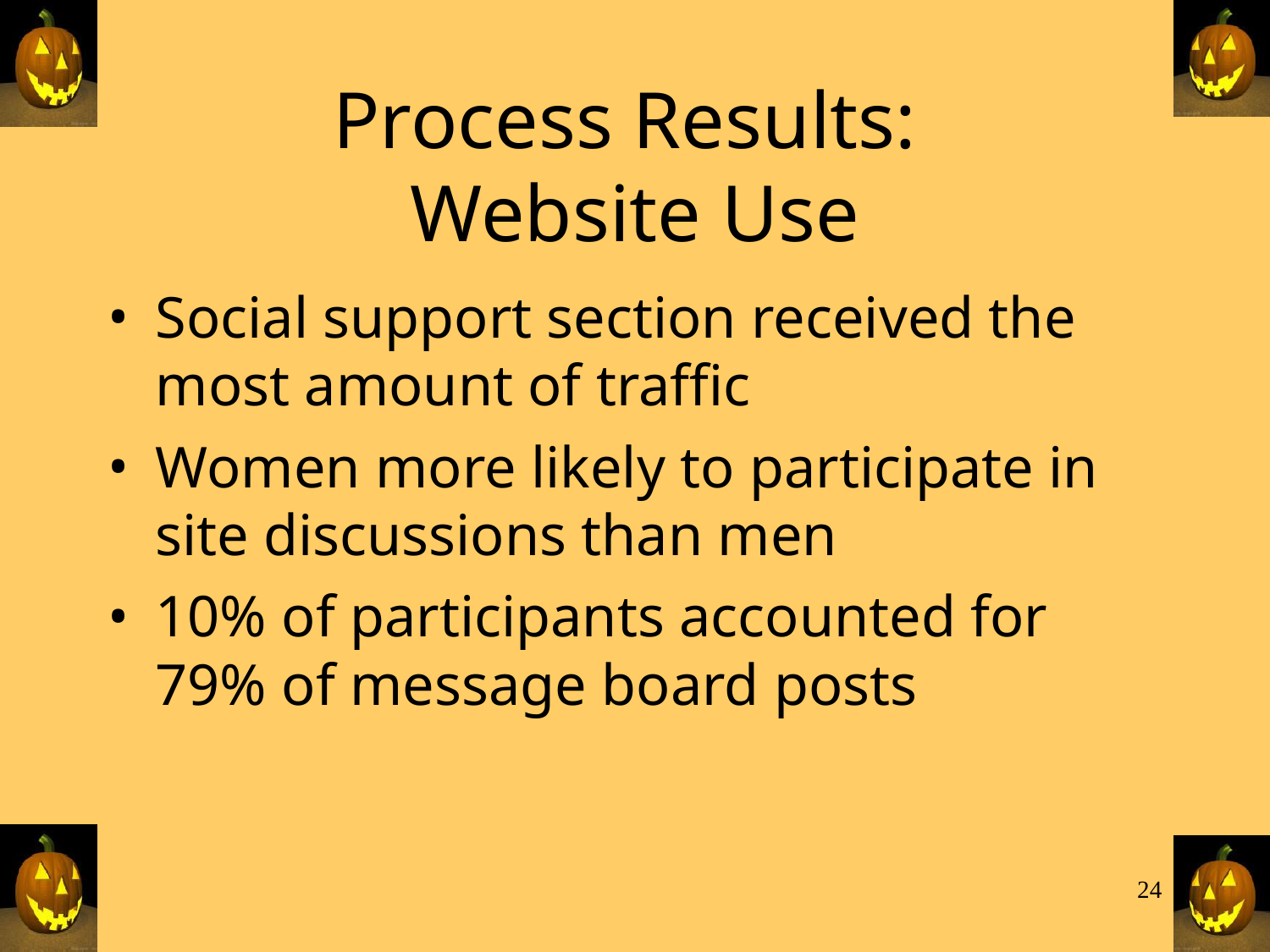

# Process Results: Website Use
Social support section received the most amount of traffic
Women more likely to participate in site discussions than men
10% of participants accounted for 79% of message board posts
24

## Slide 25
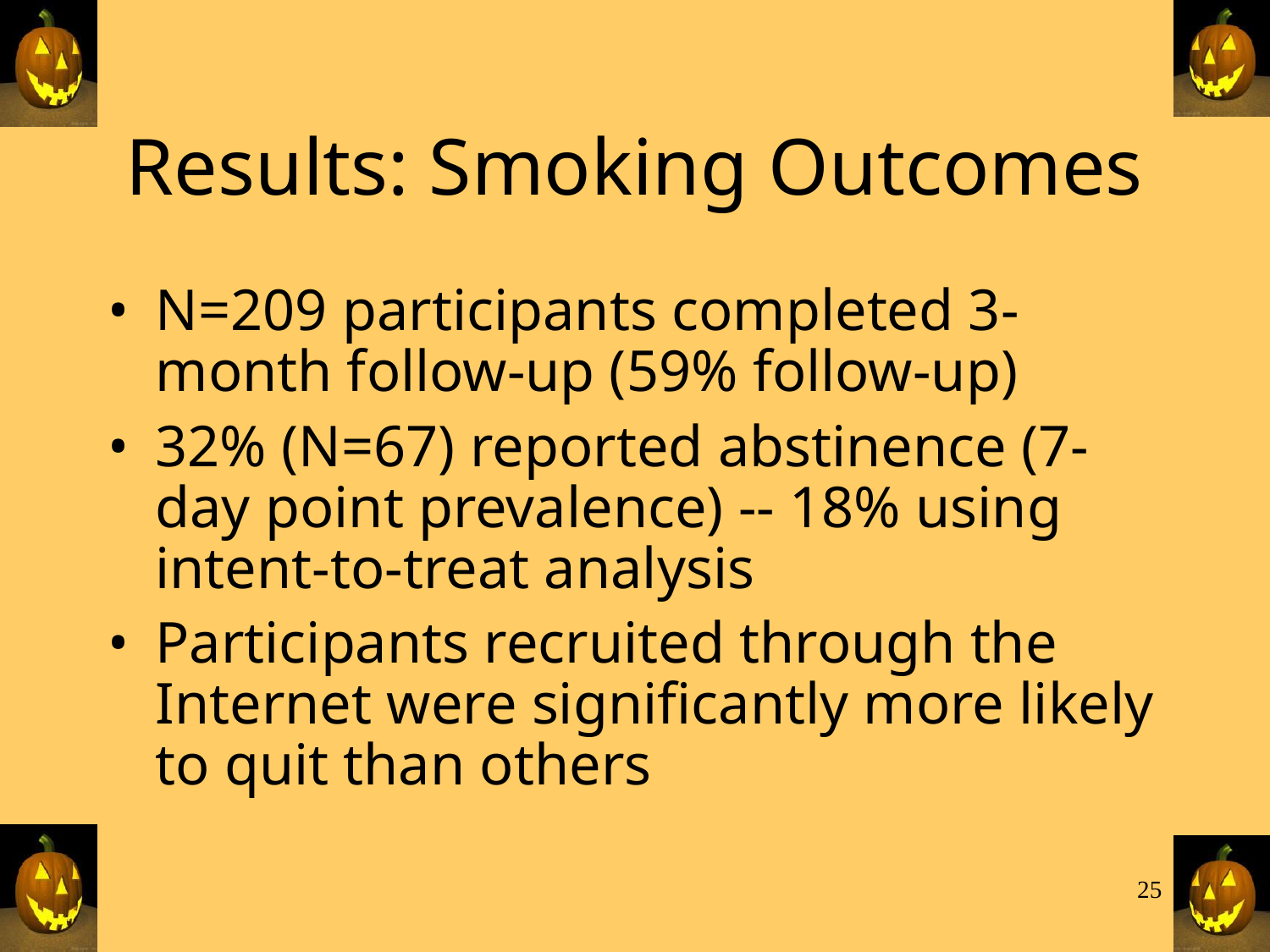

# Results: Smoking Outcomes
N=209 participants completed 3-month follow-up (59% follow-up)
32% (N=67) reported abstinence (7-day point prevalence) -- 18% using intent-to-treat analysis
Participants recruited through the Internet were significantly more likely to quit than others
25

## Slide 26
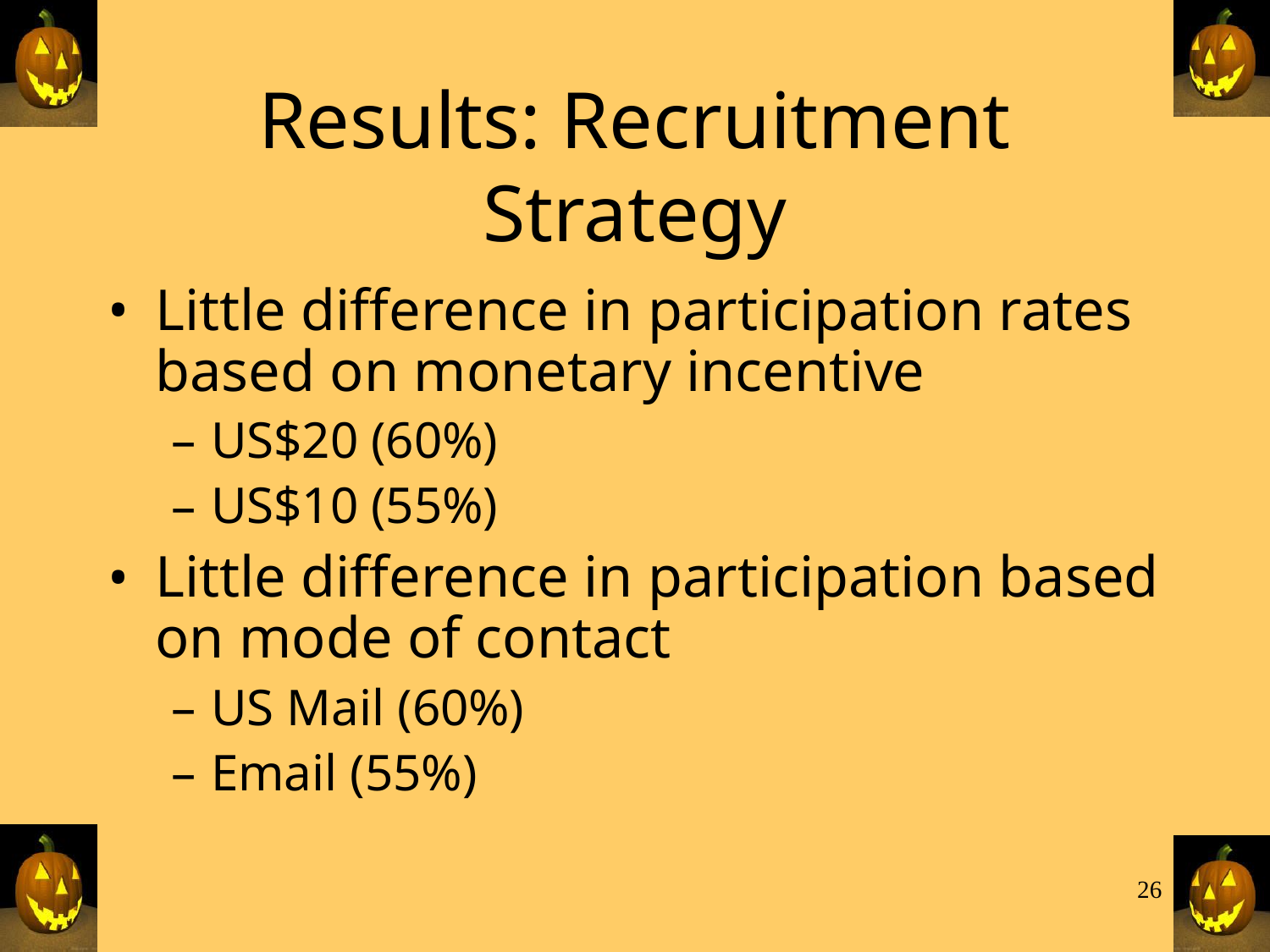

# Results: Recruitment Strategy
Little difference in participation rates based on monetary incentive
US$20 (60%)
US$10 (55%)
Little difference in participation based on mode of contact
US Mail (60%)
Email (55%)
26

## Slide 27
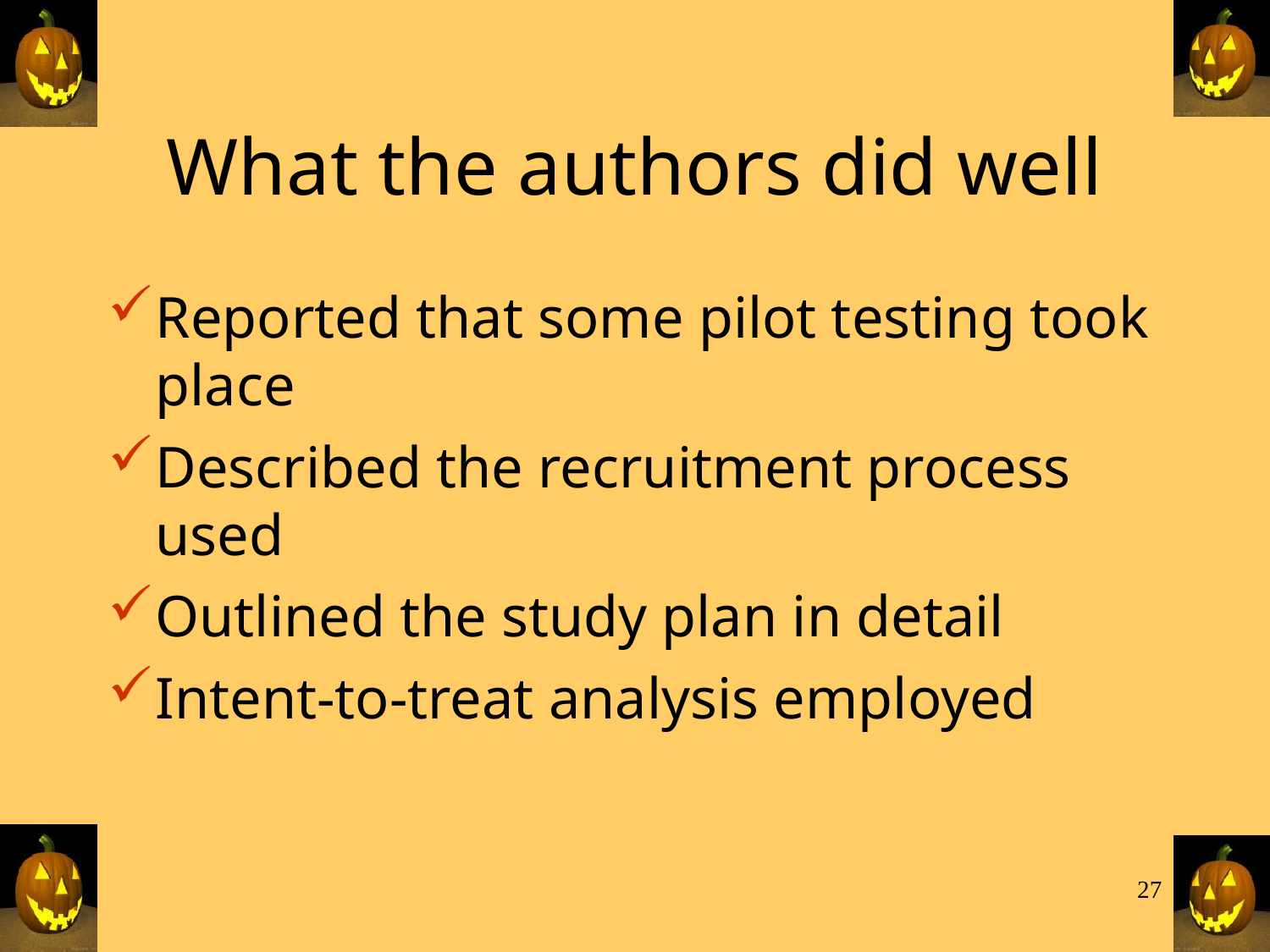

# What the authors did well
Reported that some pilot testing took place
Described the recruitment process used
Outlined the study plan in detail
Intent-to-treat analysis employed
27

## Slide 28
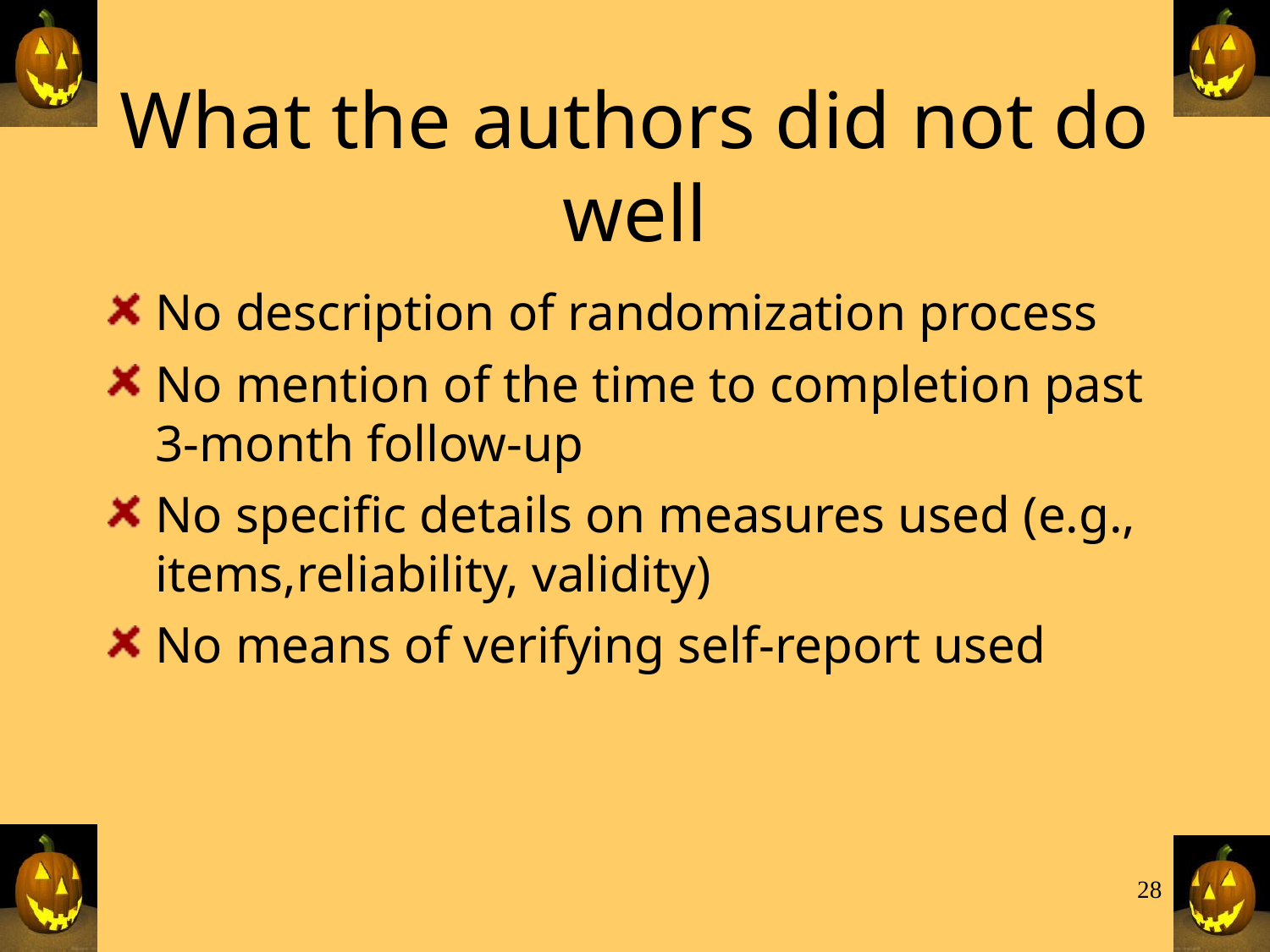

# What the authors did not do well
No description of randomization process
No mention of the time to completion past 3-month follow-up
No specific details on measures used (e.g., items,reliability, validity)
No means of verifying self-report used
28

## Slide 29
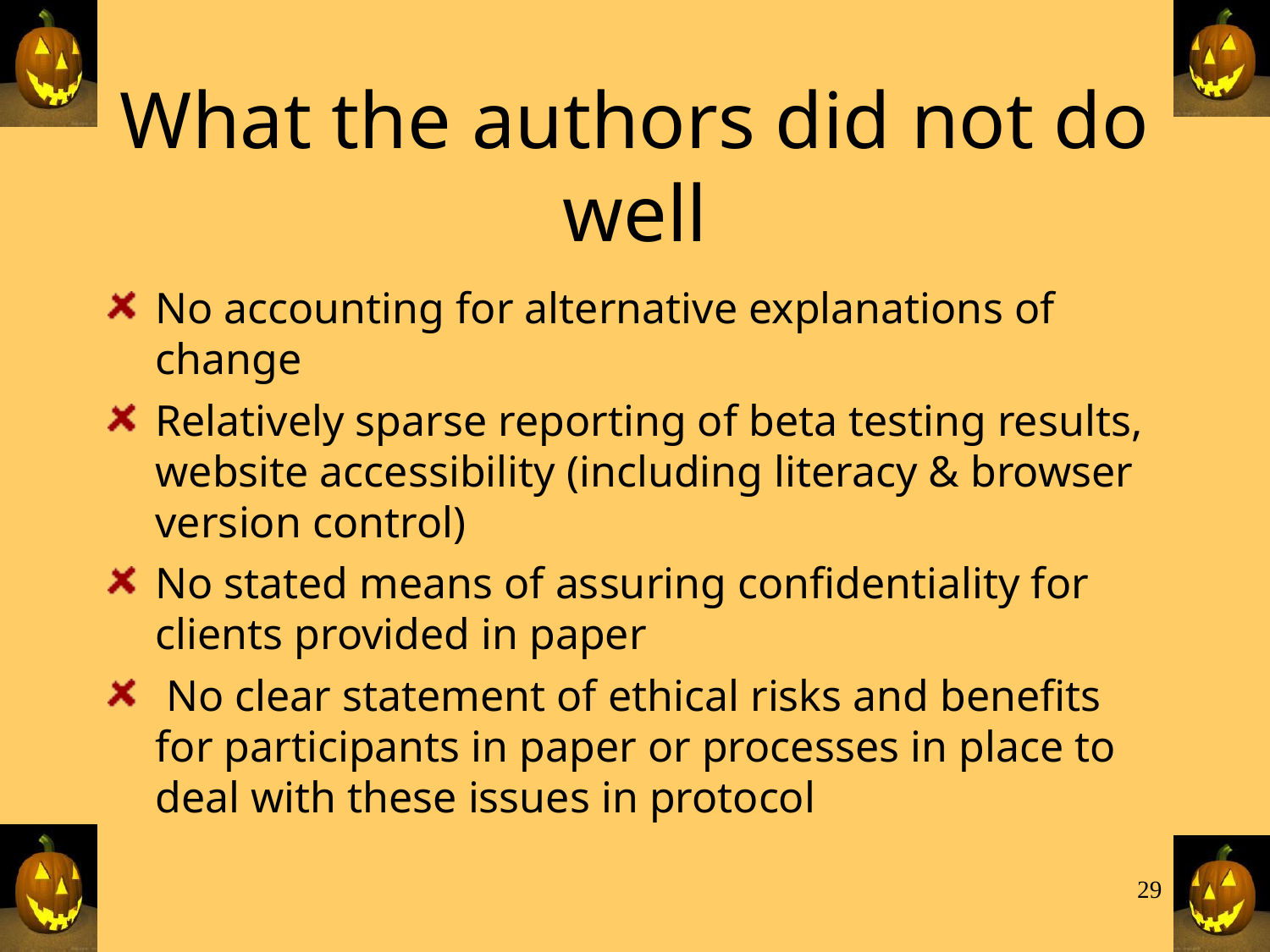

# What the authors did not do well
No accounting for alternative explanations of change
Relatively sparse reporting of beta testing results, website accessibility (including literacy & browser version control)
No stated means of assuring confidentiality for clients provided in paper
 No clear statement of ethical risks and benefits for participants in paper or processes in place to deal with these issues in protocol
29

## Slide 30
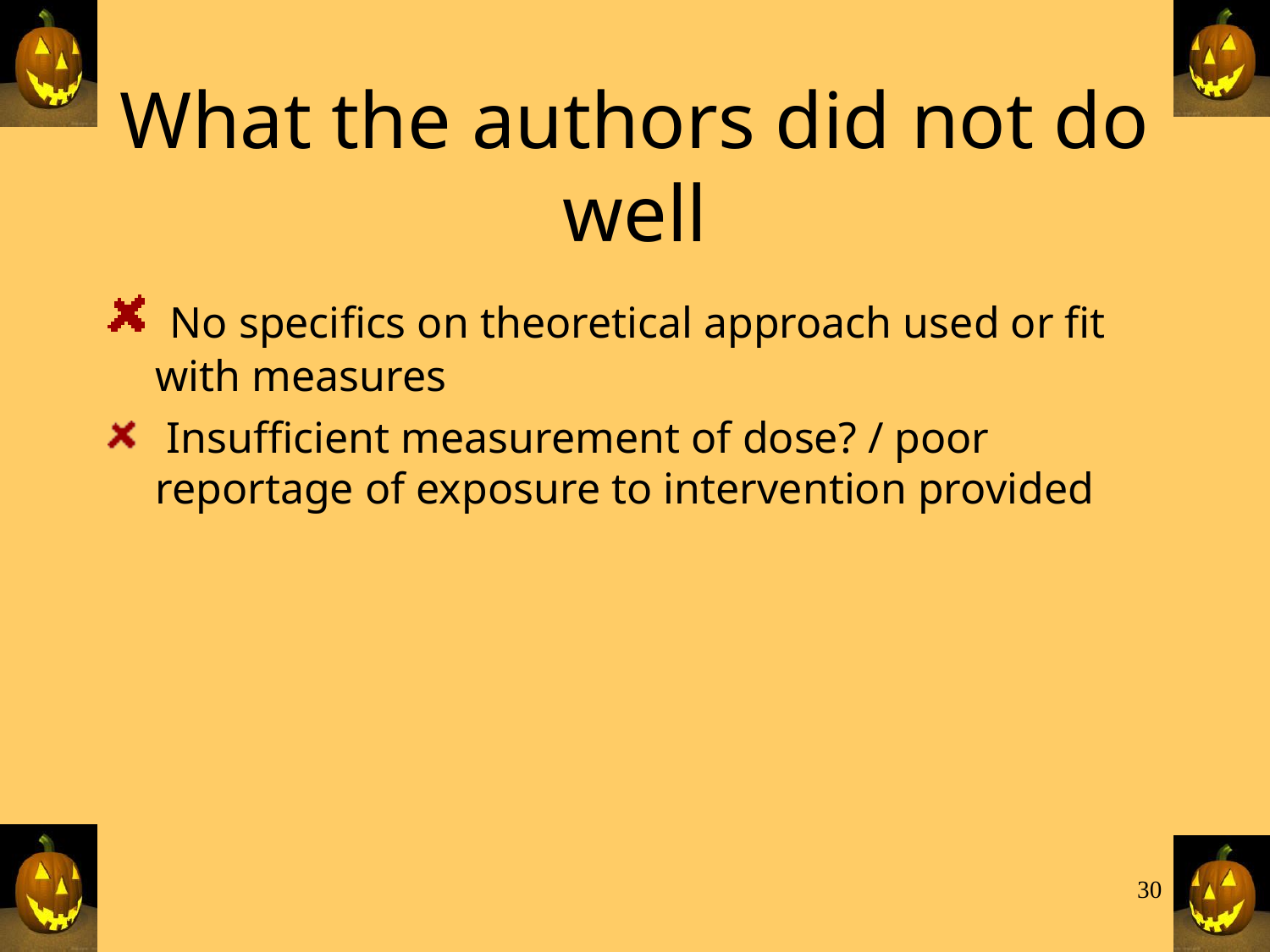

# What the authors did not do well
 No specifics on theoretical approach used or fit with measures
 Insufficient measurement of dose? / poor reportage of exposure to intervention provided
30

## Slide 31
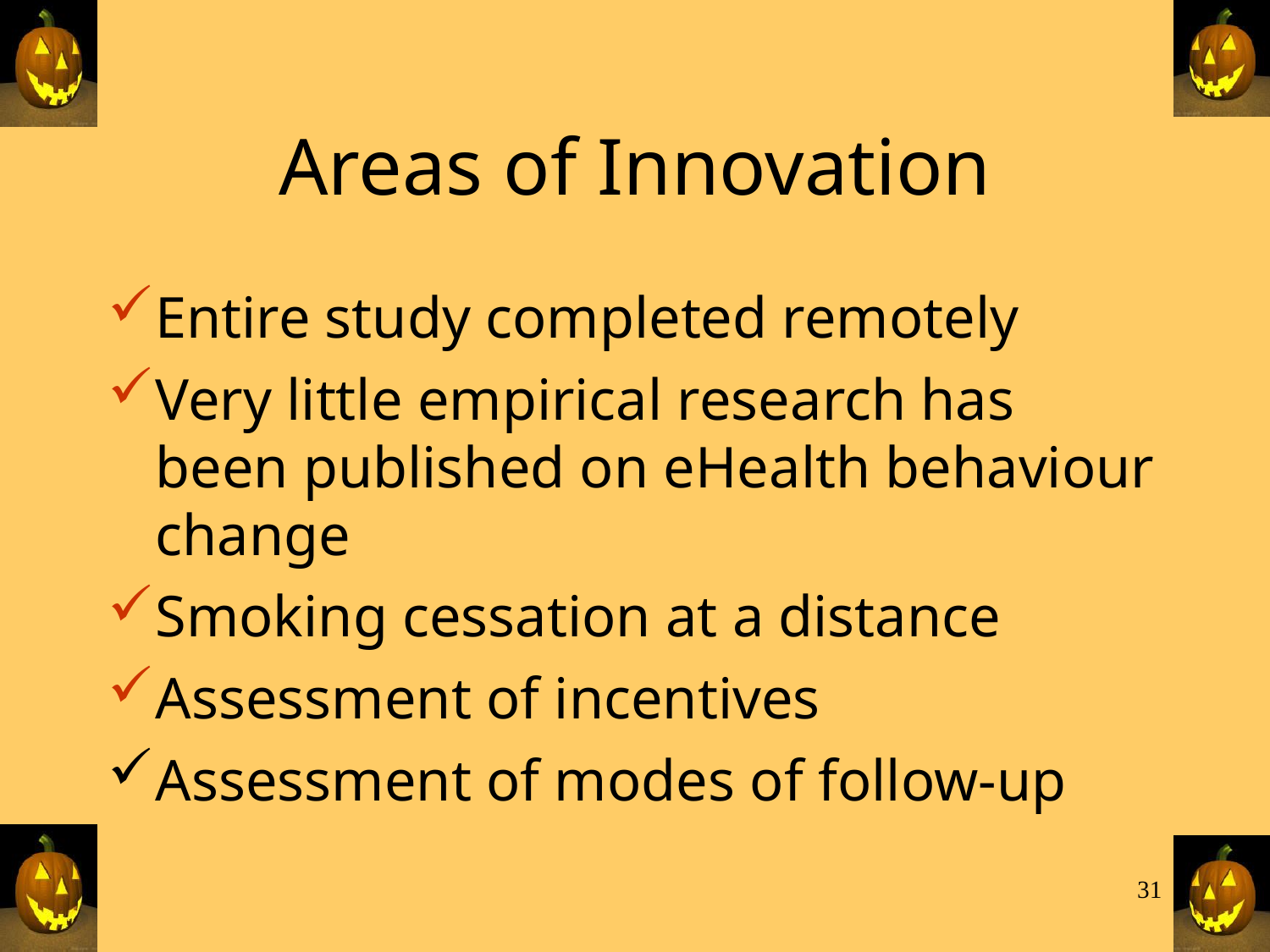

# Areas of Innovation
Entire study completed remotely
Very little empirical research has been published on eHealth behaviour change
Smoking cessation at a distance
Assessment of incentives
Assessment of modes of follow-up
31

## Slide 32
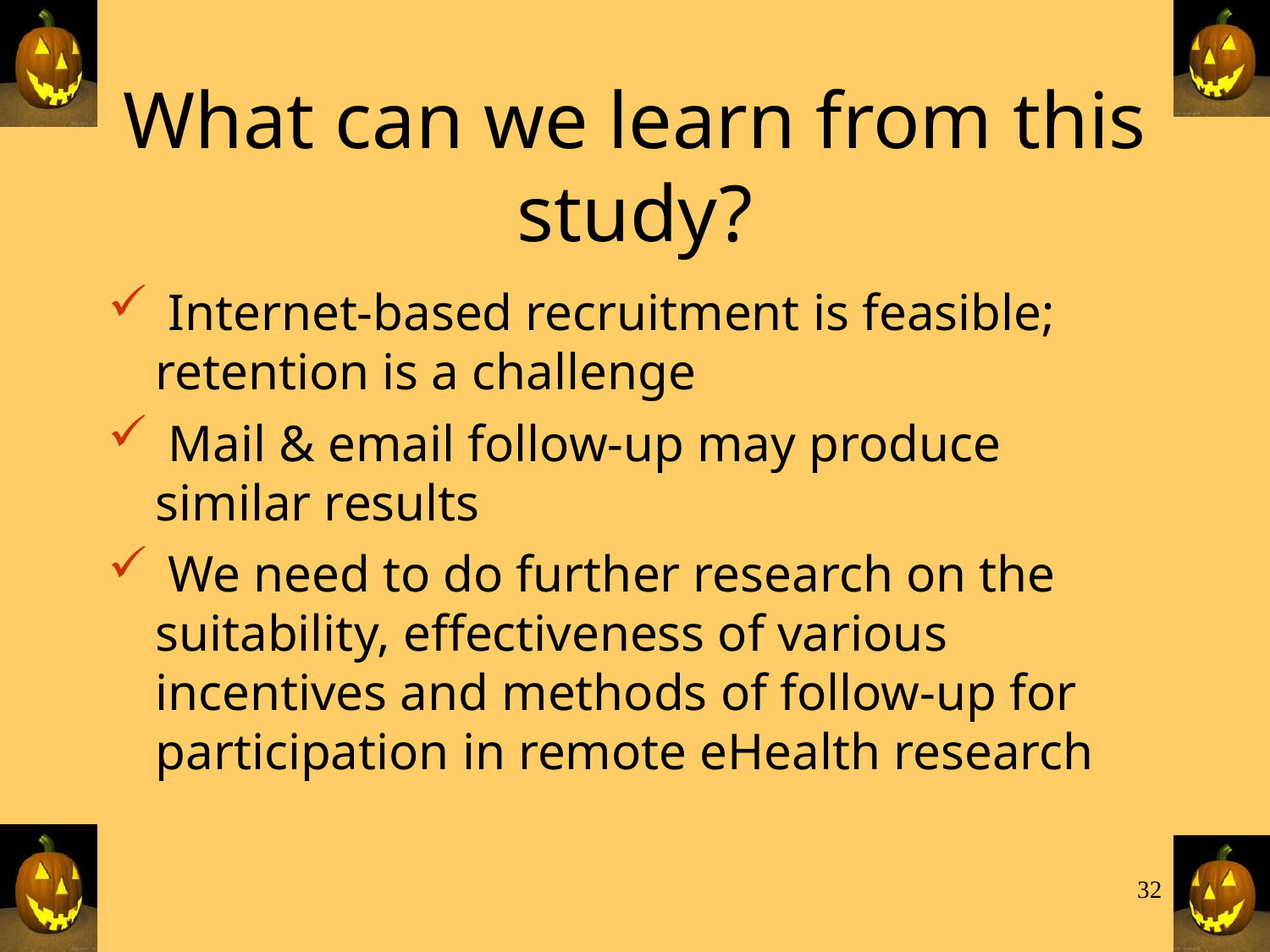

# What can we learn from this study?
 Internet-based recruitment is feasible; retention is a challenge
 Mail & email follow-up may produce similar results
 We need to do further research on the suitability, effectiveness of various incentives and methods of follow-up for participation in remote eHealth research
32

## Slide 33
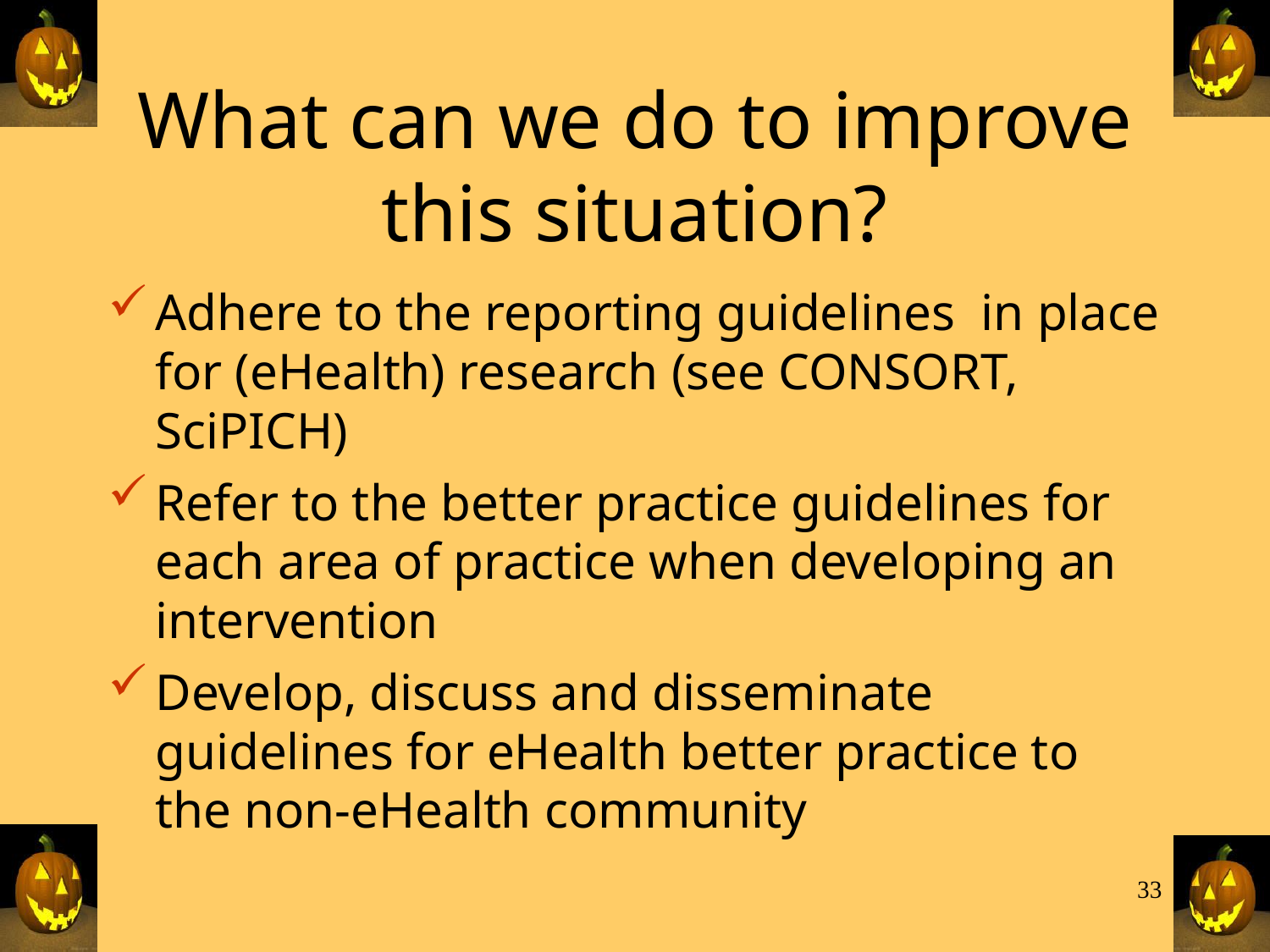

# What can we do to improve this situation?
Adhere to the reporting guidelines in place for (eHealth) research (see CONSORT, SciPICH)
Refer to the better practice guidelines for each area of practice when developing an intervention
Develop, discuss and disseminate guidelines for eHealth better practice to the non-eHealth community
33

## Slide 34
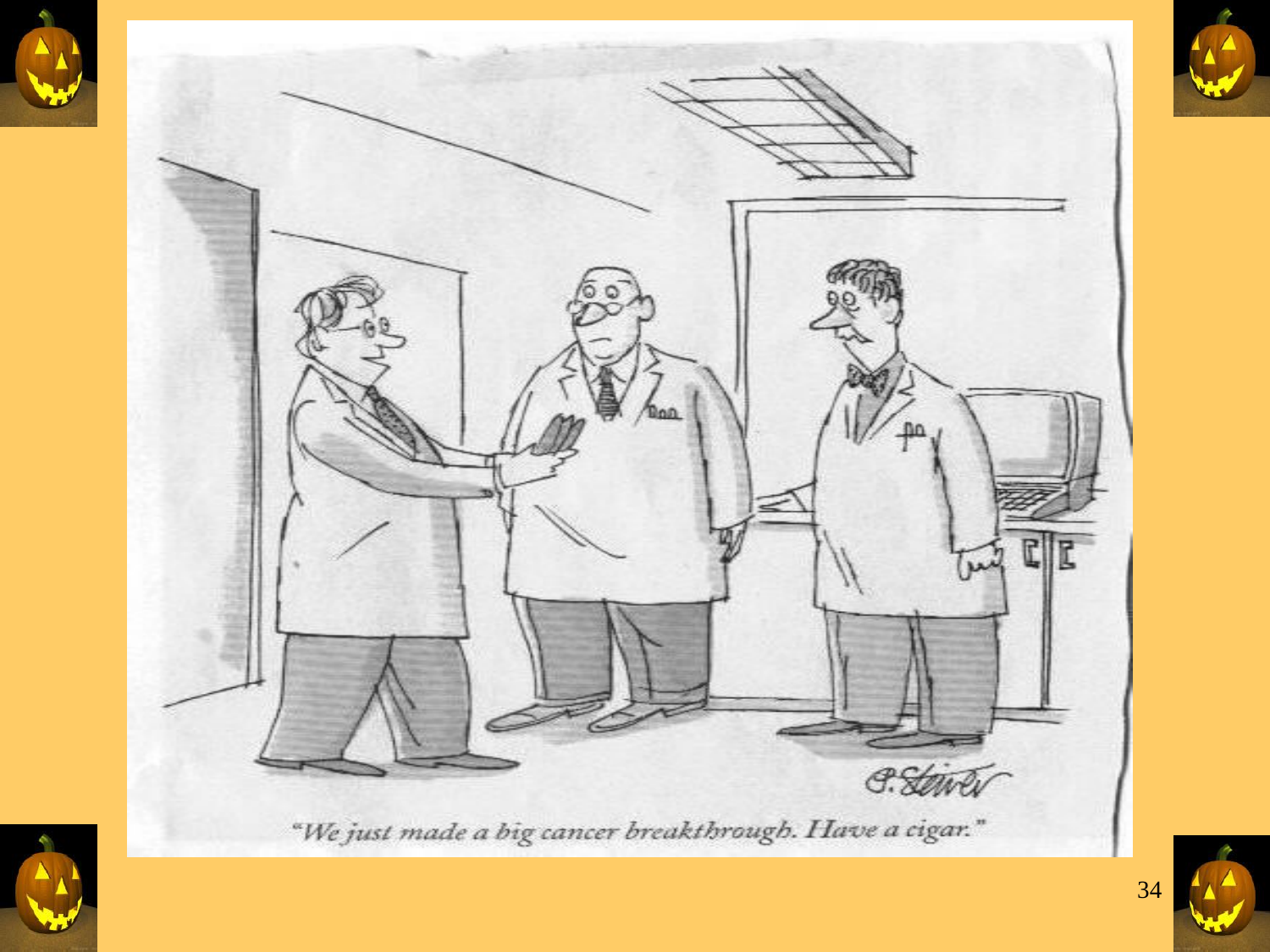

34

## Slide 35
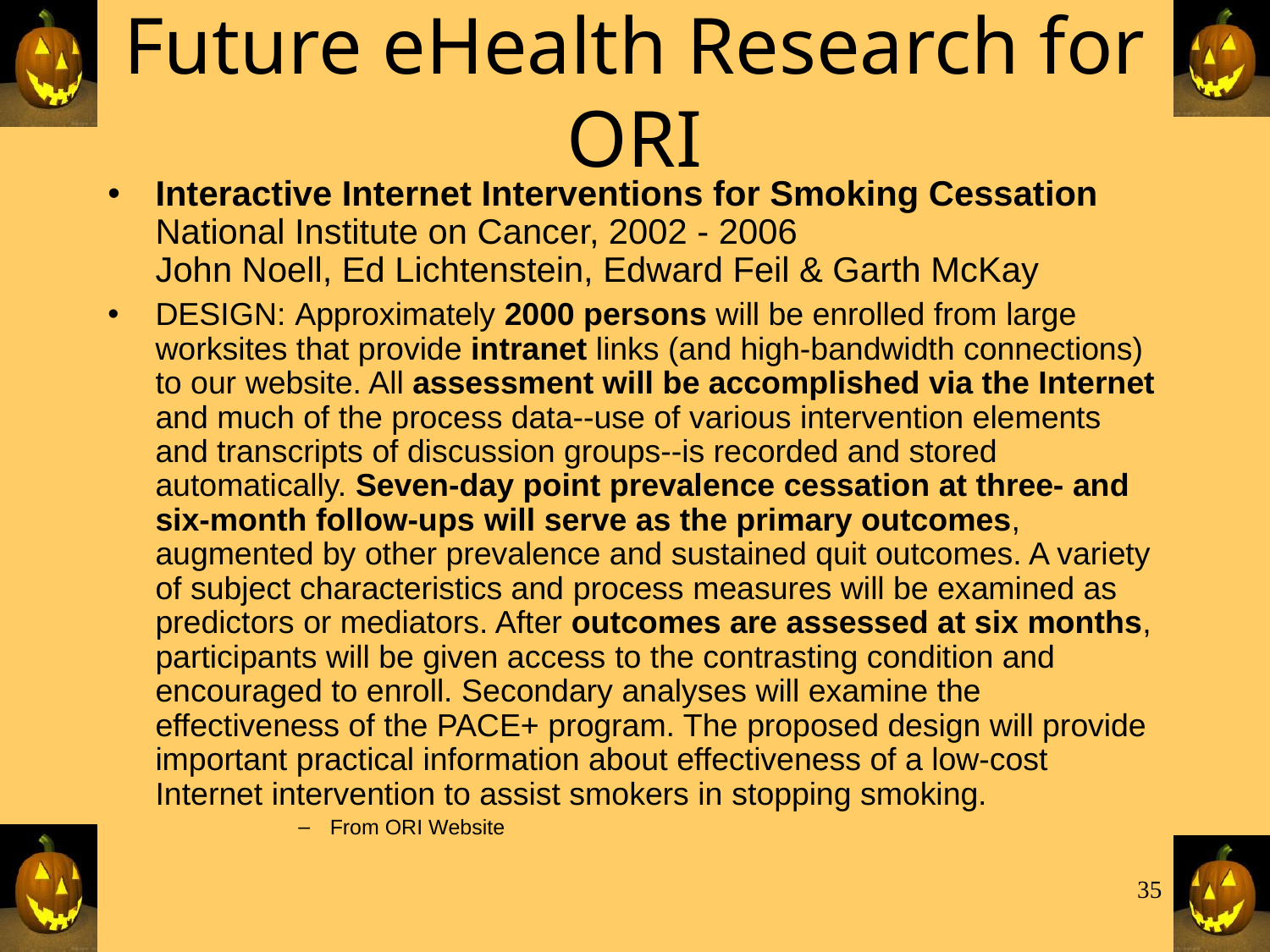

# Future eHealth Research for ORI
Interactive Internet Interventions for Smoking Cessation
National Institute on Cancer, 2002 - 2006
John Noell, Ed Lichtenstein, Edward Feil & Garth McKay
DESIGN: Approximately 2000 persons will be enrolled from large worksites that provide intranet links (and high-bandwidth connections) to our website. All assessment will be accomplished via the Internet and much of the process data--use of various intervention elements and transcripts of discussion groups--is recorded and stored automatically. Seven-day point prevalence cessation at three- and six-month follow-ups will serve as the primary outcomes, augmented by other prevalence and sustained quit outcomes. A variety of subject characteristics and process measures will be examined as predictors or mediators. After outcomes are assessed at six months, participants will be given access to the contrasting condition and encouraged to enroll. Secondary analyses will examine the effectiveness of the PACE+ program. The proposed design will provide important practical information about effectiveness of a low-cost Internet intervention to assist smokers in stopping smoking.
From ORI Website
35

## Slide 36
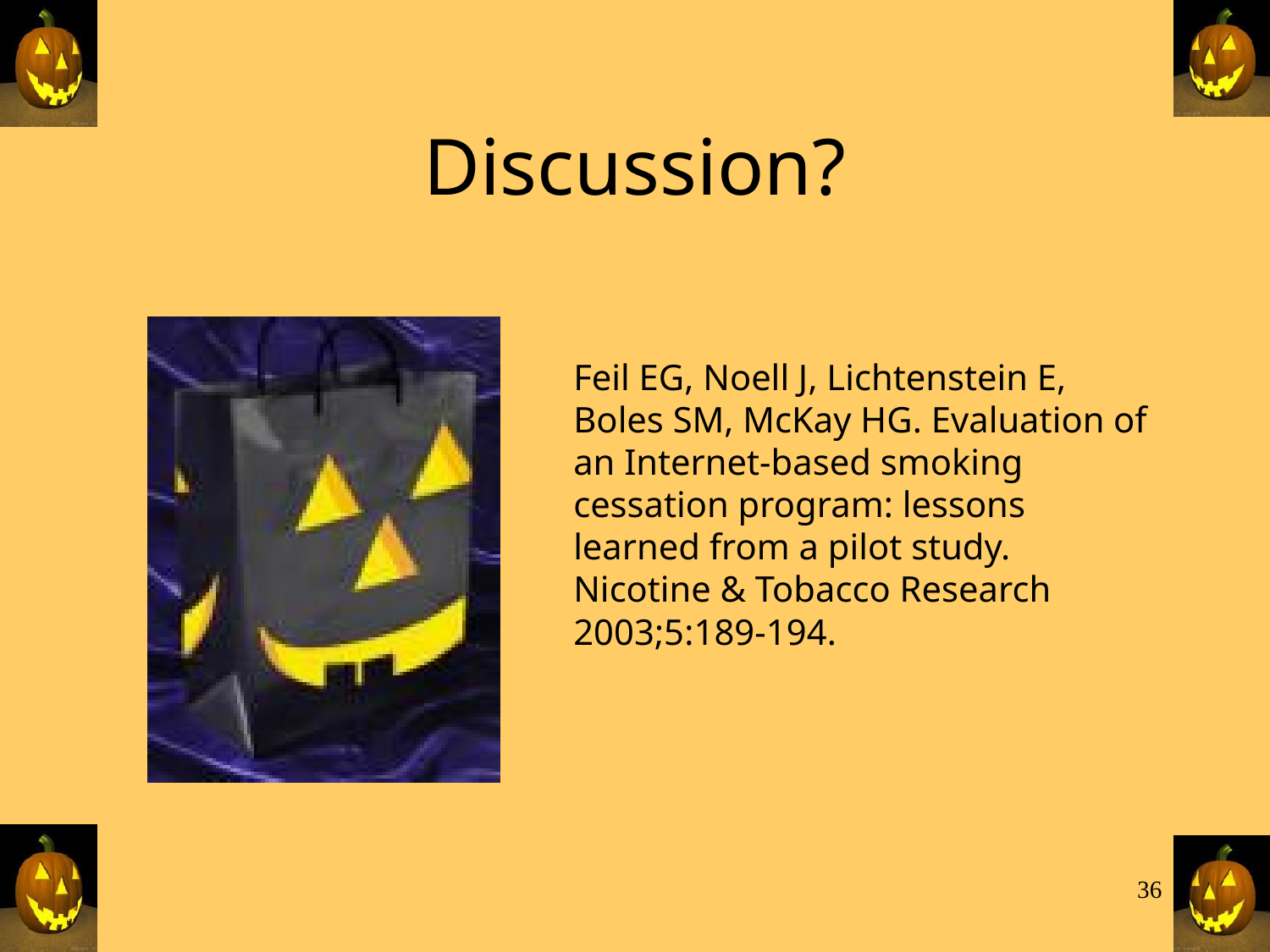

# Discussion?
Feil EG, Noell J, Lichtenstein E, Boles SM, McKay HG. Evaluation of an Internet-based smoking cessation program: lessons learned from a pilot study. Nicotine & Tobacco Research 2003;5:189-194.
36
